# Urban theft prediction via LLM-empowered spatiotemporal transformer

**DOI:** 10.1038/s41598-026-45681-0

**Published:** 2026-03-31

**Authors:** Minghu Tang, Junjie Wang, Xuan Bu, Jiayi Zhang, Peng Luo

**Affiliations:** 1 School of Intelligent Science and Engineering, Qinghai Minzu University, Xining, 810007 China; 2 School of Cyberspace Security, Qinghai Minzu University, Xining, 810007 China; 3 Joint Laboratory of Cyberspace Security, Qinghai Minzu University, Xining, 810007 China; 4 School of Computer Science and Technology, Qinghai Normal University, Xining, 810016 China

**Keywords:** Crime Prediction, Taxi Passenger Flow Proxy, Multi-Source Spatio-Temporal Feature Fusion, LLM Lightweight Fine-Tuning/Edge Fine-Tuning, Semantic-Spatio-Temporal Transformer, Engineering, Mathematics and computing

## Abstract

With the deepening of urbanization, the spatiotemporal heterogeneity of theft crimes in New York City has become prominent, creating a demand for more accurate prediction. Existing models face limitations in capturing nonlinear correlations, integrating multi-source data, and generalizing to dynamic scenarios. This study proposes an LLM-enhanced Spatiotemporal Transformer (LLM-STT) model, which integrates multi-source spatiotemporal features (including taxi passenger flow proxy) and Gemma3-12B embeddings, with a lightweight fine-tuning scheme for Gemma3-1B. Its main explorations include LLM-based semantic encoding, quantifying feature coupling, and balancing performance and deployment feasibility. Experiments on hourly neighborhood-scale theft prediction in New York City show the model achieves an AUC of 0.91 and an F1 score of 0.83, demonstrating competitive performance against baselines. LLM embeddings and dynamic population features contribute positively, and the lightweight fine-tuned model outperforms the random baseline. These findings offer preliminary support for targeted crime prevention in similar urban contexts, with broader generalization requiring further validation.

## Introduction

**Fig. 1:**
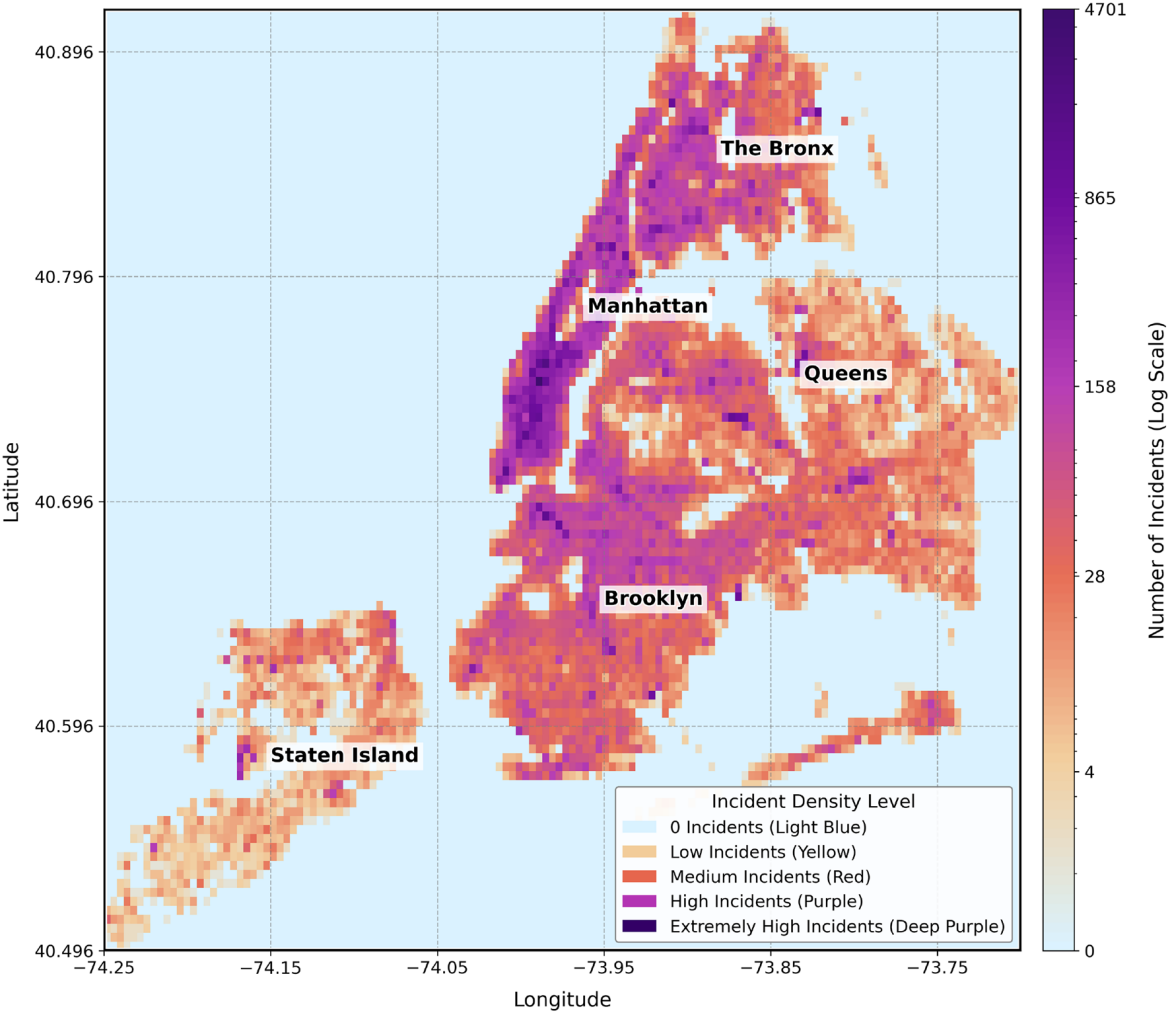
Grid density heatmap of theft cases in New York City.

With the deepening of urbanization, the spatiotemporal heterogeneity of urban theft crimes has become increasingly prominent, and accurate prediction of crime distribution has become a core issue in improving the efficiency of public safety governance. Taking New York City, a typical representative of high-density cities, as an example, its theft crimes are deeply coupled with the dynamic social environment, and the situation is severe: in 2022, the number of petty theft cases in the city increased by 33.9%, the number of motor vehicle thefts exceeded 13,000 (a 16-year high), and large retailers suffered losses of more than 500 million US dollars due to theft ; approximately 65% of inventory losses in the US retail industry in 2022 were caused by theft , with theft risks in core cities such as New York significantly higher than the national average. From the perspective of spatial distribution (see Fig. 1), theft crimes in New York are highly concentrated in dense commercial areas such as Midtown Manhattan^1^. The difference in case density at the grid scale (approximately 14.3 hectares) reaches hundreds of times^2^, highlighting the dual predictive challenges of “spatial heterogeneity + dynamic risks”^3^.

Recent advancements in deep learning have significantly reshaped the landscape of spatiotemporal crime prediction, evolving from conventional convolutional and recurrent architectures to more sophisticated paradigms that better capture the intricate dynamics of urban crimes. In the realm of graph-based methodologies, researchers have moved beyond simple Graph Convolutional Networks (GCNs) to address issues of heterogeneity and generalizability. For instance, frameworks like EX-Crime leverage the Graph Information Bottleneck principle to extract type-aware periodic patterns, enhancing model explainability^4^. Similarly, the Ada-GCNLSTM model integrates an adaptive Maximum Mean Discrepancy (MMD) mechanism with GCNs and Long Short-Term Memory (LSTM) networks to filter noise and capture latent correlations across different crime categories, thereby improving robustness and generalization^5^. Furthermore, multi-scale architectures such as ST-HGNet have been proposed to tackle the sparsity of crime data by employing hierarchical gating structures to enhance feature perception at various spatial resolutions^6^. The push for generalizability has also led to the development of unified frameworks like the attention-based GCN-LSTM, which can adapt to diverse urban prediction tasks without architectural modification by fusing spatial, temporal, and static features through a learnable gating mechanism^7^.

Parallel to the evolution of GNNs, attention mechanisms and Transformer architectures have emerged as powerful tools for modeling the complex dependencies inherent in crime data. Models like the Neural Attentive framework (NAHC) demonstrate the efficacy of fusing spatial, temporal, and categorical information for high-resolution, hour-level crime prediction, effectively addressing the issue of data sparsity through prior-knowledge augmentation^8^. To handle the non-stationarity of crime time series, the START model combines a spatiotemporal Transformer with a vector autoregressive component, enabling more precise forecasting by focusing on specific crime-type information^9^. Recognizing the computational demands of Transformers, recent research has also focused on efficiency; for example, ACSAformer introduces a sparse attention mechanism to reduce computational complexity^10^, while lightweight hybrids like LCRNet, which integrates Transformers with CNNs and a simulated annealing sparsity mechanism, achieve high accuracy with significantly reduced FLOPs, making them suitable for edge deployment^11^. Beyond improving prediction accuracy alone, a growing body of research is now exploring how to bridge the gap between prediction and actionable interventions. For example, the ICON framework integrates PredRNN++ for spatiotemporal forecasting with constraint programming to form a closed prediction-action loop, demonstrating how deep learning predictions can directly inform and optimize police patrol routes^12^. This shift from pure prediction to decision optimization represents a critical step toward operationalizing crime forecasting in real-world policing contexts.

The integration of recurrent architectures with pre-trained models further opens new frontiers. The combination of ST-ResNet and LSTM has been shown to overcome the limitations of treating time as parallel channels by explicitly modeling sequential dependencies^13^. More innovatively, frameworks like CriX are beginning to leverage pre-trained language models such as InLegalBERT and Large Language Models (LLMs) combined with Retrieval-Augmented Generation (RAG) to generate not only predictions but also interpretable textual justifications, marking a significant step towards explainable and knowledge-infused crime analysis^14^.

Although existing research on theft crime prediction has undergone multi-stage development, it still has insurmountable systematic limitations: in terms of model capability, traditional statistical models (such as logistic regression, GBDT) cannot capture the nonlinear correlations between “peak passenger flow and crime opportunities”^15^; although spatiotemporal deep learning models (such as ST-ResNet, ST-SHN) can handle temporal dependencies, they are limited by static spatial assumptions, and their prediction errors for taxi passenger flow proxy scenarios (such as morning and evening commutes, temporary gatherings during holidays) are only 4.7% lower than those of static models^16,17^ ; in terms of data fusion, existing methods mostly rely on structured data (such as historical crime records, number of POIs) and have the problem of “semantic fragmentation of multi-source data”–for example, using only POI categories cannot distinguish the risk differences between “mall opening and mall closure”, and nighttime light data fails to be deeply coupled with venue functions (such as commercial/residential), resulting in a prediction lag of 2-3 weeks for emerging commercial areas; in terms of knowledge reasoning, current models are generally driven by historical data, which not only lack an understanding of scenario-specific semantics such as “high nighttime risks in commercial dense areas” but also tend to learn historical law enforcement biases. Their generalization ability decreases significantly when facing abrupt changes in population flow (such as large-scale events, extreme weather). The British police once failed to implement similar prediction models in practice due to their low accuracy and ethical controversies^18^.

To address the aforementioned limitations, this study proposes an LLM-Enhanced Spatiotemporal Transformer (LLM-STT) model that integrates multi-source spatiotemporal features (including taxi passenger flow proxy) and Large Language Model (LLM) embeddings^19,20^. This model is aimed at achieving hourly high-resolution prediction at the neighborhood scale in New York City, which covers a 106$\times$139 grid with approximately 14.3 hectares per grid. The specific research objectives are as follows: (1) Quantify the temporal patterns of taxi passenger flow proxy based on taxi trajectories, integrate multi-source data such as Points of Interest (POI), nighttime light, and meteorological data, and break free from the reliance of traditional feature engineering on structured data; (2) Generate semantic scores for crime risk using an LLM (Gemma3-12B), and combine with BERT semantic encoding to realize the dual-source fusion of “data-driven + automatic feature crossing”;(3) Enhance prediction reliability in high-risk areas through multi-source semantic fusion, thereby offering theoretical and technical references for targeted theft crime prevention and control in similar high-density urban contexts, with broader applicability requiring further validation across diverse scenarios.

## Data and methods

### Study area and data

The study area is New York City, USA, with a geographical scope covering North Latitude 40.496$^{\circ }$-40.915$^{\circ }$ and West Longitude 73.7$^{\circ }$-74.25$^{\circ }$, including five boroughs: Manhattan, Brooklyn, Queens, the Bronx, and Staten Island. This study integrates 5 categories of core data, all of which have undergone data cleaning (removing outliers and imputing missing values) and spatiotemporal alignment (matching grids and time windows). The specific information is shown in the table 1:

**Table 1:** Sources, Time Ranges, and Purposes of Core Data for Theft Crime Prediction in New York City.

Data Type	Source	Time Range	Core Content
Crime Data	New York City Police Department Open Database	Nov 2013–Dec 2015, Jan 2020–Dec 2020	539,872 theft case records (2013–2015) and 121,659 records (2020) (including occurrence time and coordinates). 619,059 cleaned records used as labels (1 = crime, 0 = no crime).
Taxi Passenger Flow Proxy Data	New York City Taxi Trajectory Dataset	Jan 2013–Dec 2015, Jan 2020–Dec 2020	Hourly grid-level inflow (destination passengers), outflow (origin passengers), and net flow to measure population aggregation intensity.
Multi-Source Spatiotemporal Data	NOAA-VIIRS	Jan 2013–Dec 2015, Jan 2020–Dec 2020	Nighttime light data (500m resolution) for regional activity intensity.
	OpenStreetMap (POI)	-	12 POI categories (commercial, residential, etc.) for functional density and road network accessibility.
Auxiliary Control Data	National Weather Service (U.S.)	Nov 2013–Dec 2015, Jan 2020–Dec 2020	Meteorological data to control environmental interference.
	U.S. Census Bureau	-	Socio-economic data (GDP, population density).

### Overall model framework

**Fig. 2:**
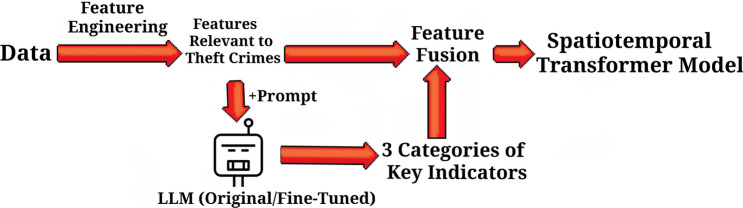
Schematic diagram of the model’s technical flow.

For the task of predicting theft crimes in New York City, this study constructs an LLM-Enhanced Spatiotemporal Transformer (LLM-STT) model, aiming to achieve cross-modal fusion of dynamic population, multi-source spatiotemporal features, and LLM semantics. The specific technical route is as follows: first, extract the relevant influencing factors of theft crimes through feature engineering; then input the above-mentioned features into the large language model (LLM), and rely on prompt guidance and fine-tuning strategies to prompt the model to output 3 types of key indicators by combining its own reasoning capabilities and knowledge reserves; finally, fuse all features with the 3 types of indicators output by the LLM to construct a feature set, and use the spatiotemporal Transformer model to conduct training. The specific process is shown in Fig. 2.

#### Overall model architecture, inputs and outputs

The input of the model is the feature set $\textbf{X}_{i,t} = \{X_{\text {temporal}}, X_{\text {spatial}}, X_{\text {poi}}, X_{\text {flow}}, X_{\text {llm}}\}$ of grid *i* at time *t*, including 5 categories of features: temporal features (such as seasonal coefficients, $d_t = 6$), spatial features (such as road network density, $d_s = 8$), venue functional features (such as commercial density, $d_p = 5$), taxi passenger flow proxy features (such as inflow volume, $d_f = 7$), and LLM semantic features (such as semantic risk scores, $d_l = 771$); the output is the crime occurrence probability of grid *i* at time *t*, $P(y_{i,t} = 1 \mid \textbf{X}_{i,t}) \in \{0, 1\}$.

#### Design of core modules

Design of Core Modules The core architecture of the spatiotemporal Transformer model is divided into 4 layers, which are detailed as follows: Design of Core Modules The core architecture of the spatiotemporal Transformer model is divided into 4 layers, which are detailed as follows:

(1) Feature Encoding Layer: For the unified mapping of heterogeneous features, type-aware encoding is used to eliminate dimension differences and map different types of features to the same semantic space (hidden layer dimension $d_h=256$): continuous features (such as inflow volume, nighttime light intensity) are processed through linear transformation and LeakyReLU activation; discrete features (such as weather type, POI function) are converted into vectors via an embedding layer; LLM semantic features (771-dimensional) are aligned with other features through dimension reduction (where $W_{\text {llm}} \in \mathbb {R}^{256 \times 771}$); finally, the initial feature $Z_0$ is formed by integration through layer normalization^21^ .

(2) Spatiotemporal Attention Interaction Layer: To capture dynamic dependencies, a dual-attention module is designed to quantify spatiotemporal correlations, adapting to the spatial spillover and temporal dynamics of crime risks: spatial attention takes the 2nd-order neighborhood (36 grids) as the scope, calculates interaction weights between grids based on latitude and longitude differences^22^ , and generates spatial context features $Z_{t,i}^{\text {spatial}}$, which reflects the principle that “the closer the distance, the stronger the risk correlation”; temporal attention captures temporal dependencies between the current moment and the previous 3 hours, and generates temporal context features $Z_{t,i}^{\text {temporal}}$ by weighting according to time differences; spatiotemporal features are fused through a gating mechanism with a learnable weight $\gamma \in [0,1]$, and the output is:

1 $$\begin{aligned} Z_{t,i}^1 = \text {LayerNorm}\left( Z_{t,i} + \gamma \cdot Z_{t,i}^{\text {spatial}} + (1-\gamma ) \cdot Z_{t,i}^{\text {temporal}}\right) \end{aligned}$$

(3) LLM-Deep Learning Fusion Layer: Prior Correction Enhancement. Combining the knowledge reasoning capability of LLM and the spatiotemporal modeling advantage of deep learning, a “prior correction” mechanism is designed: the semantic risk score $P_{\text {llm},i,t}$ output by Gemma3-12B is dynamically calibrated using the formula $\hat{P}_{\text {llm},i,t} = P_{\text {llm},i,t} \cdot \sigma \left( W_p \cdot Z_{t,i}^1 + b_p\right)$ to eliminate systematic biases; the calibrated probability is subjected to element-wise interaction with $Z_{t,i}^1$ to strengthen the correlation between “high prior risk and high spatiotemporal features”, and the output is $Z_{t,i}^2$. (4) Prediction Output Layer: Probability Calculation and Loss Optimization. The final crime probability is output through a two-layer fully connected network (with LeakyReLU activation):

2 $$\begin{aligned} P(y_{i,t}=1) = \sigma \left( W_2 \cdot \text {LeakyReLU}\left( W_1 \cdot Z_{t,i}^2 + b_1\right) + b_2\right) \end{aligned}$$

The weighted cross-entropy loss (with positive sample weight $\omega =0.6$) is adopted to balance the sample distribution and strengthen the penalty for prediction errors in high-risk areas.

### Feature engineering

Based on the research objectives and data characteristics, the feature engineering system is divided into four basic feature modules: temporal features, geospatial features, venue functional features, and taxi passenger flow proxy features. It is combined with large language model (LLM) embeddings and semantic feature generation to achieve feature enhancement, forming a three-level architecture of “basic features - dynamic features - semantic features”, which is detailed as follows.

#### Temporal features

**Fig. 3:**
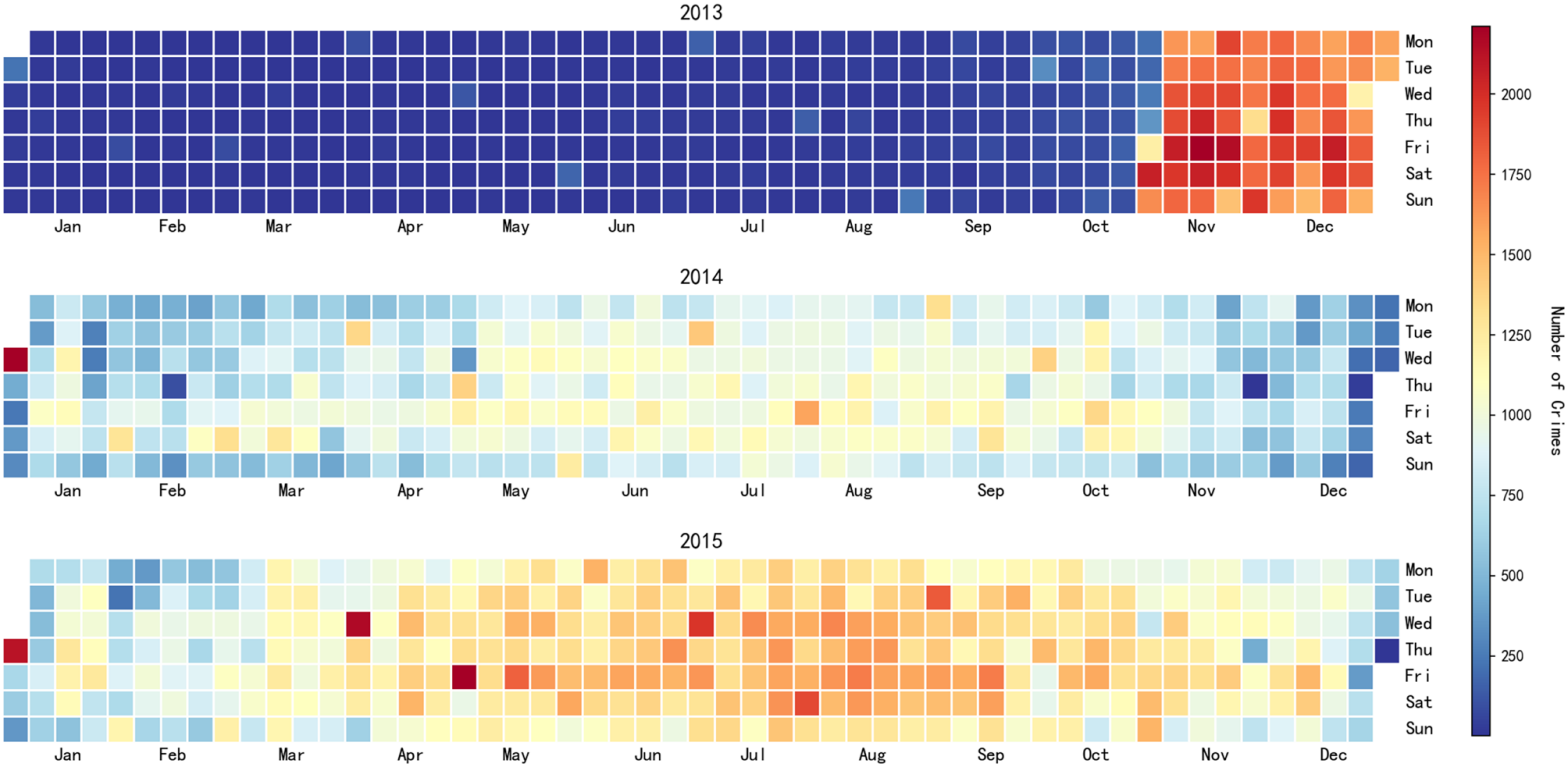
Monthly distribution heatmap of theft cases in New York City (2013–2015).

**Fig. 4:**
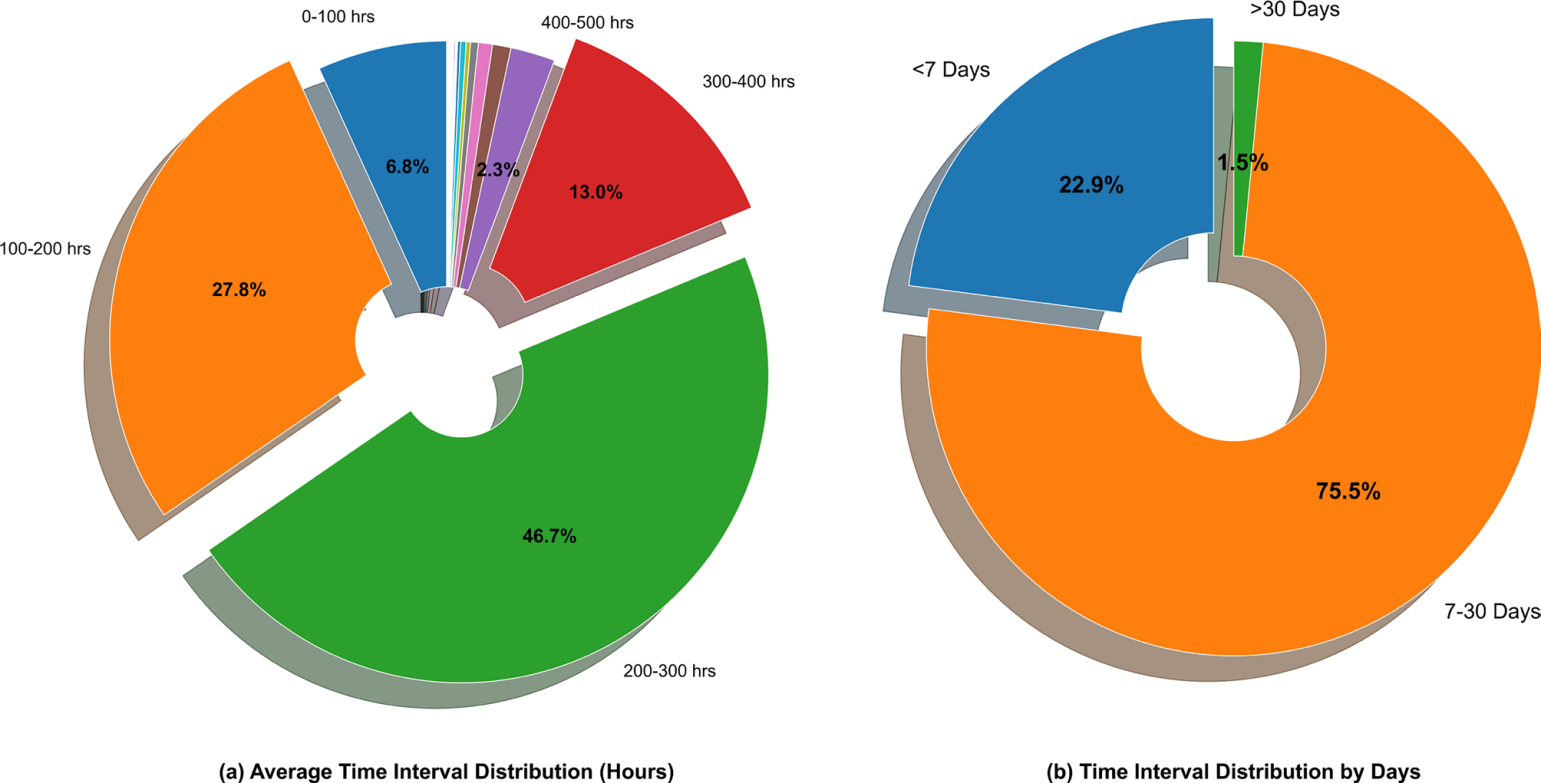
Pie Chart of the Distribution of Average Crime Time Intervals in Grid Areas of New York City.

The core goal of temporal features is to quantify the dynamic correlation between “time and crime risk”^23^. In combination with the temporal distribution pattern of theft crimes in New York City (see Figs. 3 and 4), three types of key features are extracted, which are detailed as follows:

(1) Seasonal Coefficient ($S_m$): Based on the monthly average number of crimes throughout the year, it represents the relative level of monthly crime intensity (a value of $S_m > 1$ indicates a high-risk month).

3 $$\begin{aligned} S_m = \frac{N_m}{\bar{N}} \end{aligned}$$

Where $N_m$ is the total number of crimes in the *m*-th month, and $\bar{N}$ is the monthly average number of crimes throughout the year. Statistics show that the number of crimes in New York City from April to October (warm season) is 19.3% higher than that from November to March (cold season), with secondary peaks in July-August (peak summer tourism season) and December (peak holiday shopping season). This confirms the rule that “the higher the frequency of human activities and the more aggregated scenarios in a specific season, the more crime opportunities there are”.

(2) Average Crime Time Interval ($A_i$): It calculates the average time interval between consecutive crime events within grid *i* (unit: days) and is used to distinguish the heterogeneity of regional crime frequency.

4 $$\begin{aligned} A_i = \frac{\sum _{k=2}^{K_i} \left( t_{i,k} - t_{i,k-1} \right) }{K_i - 1} \times \frac{1}{24} \end{aligned}$$

Where $K_i$ is the total number of crimes in grid *i*, and $t_{i,k}$ is the timestamp of the *k*-th crime (unit: hours); the result is converted to “days” to unify the scale. If $K_i = 1$ (only one crime occurred), $A_i$ is assigned the average crime interval of the administrative district where the grid is located (to avoid a zero denominator). As shown in Fig. 4, 75.52% of the grids in New York City have a crime interval of 7-30 days, 1.53% of the grids have an interval exceeding 30 days, and 22.94% of the grids have an interval of less than 7 days. A typical example is that for the grid (66,64) in the Manhattan commercial area, $A_i = 0.21$ days (high frequency), while for some residential grids such as grid (74,71), $A_i = 420.92$ days (low frequency). This reflects the regional heterogeneity of theft behaviors, and this feature increases the prediction sensitivity of high-frequency crime areas by 23.61%^24,25^.

(3) Risk Trigger Index ($P_{i,t}$): It combines the “time since the most recent crime ($B_{i,t}$), which refers to the time difference between the current moment and the most recent crime in grid *i*” and $A_i$, and quantifies the degree of risk accumulation through the formula: $P_{i,t} = \max \left( 0, B_{i,t} - A_i\right)$. A larger value indicates that the current risk exceeds the inherent frequency level of the region. For example, when $A_i = 10$ days and $B_{i,t} = 15$ days, $P_{i,t} = 5$, which suggests that the risk of recurrent crimes in this region in the short term increases significantly^24,25^.

All temporal features are normalized to the interval [0, 1] using Min-Max normalization to eliminate dimension differences and adapt to the input requirements of subsequent models.

#### Geospatial features

(1) Venue Functional Risk Weight (Feature C): Based on 12 categories of POI functions (Residential T1, Commercial and Retail T3, etc.) from OpenStreetMap, it is defined as the ratio of the total number of crimes in a certain type of functional area to the average area of that type of functional area^26^:

5 $$\begin{aligned} C_{i,j} = \frac{N_{\text {crime},i,j}}{\bar{S}_j} \times 100 \end{aligned}$$

**Fig. 5:**
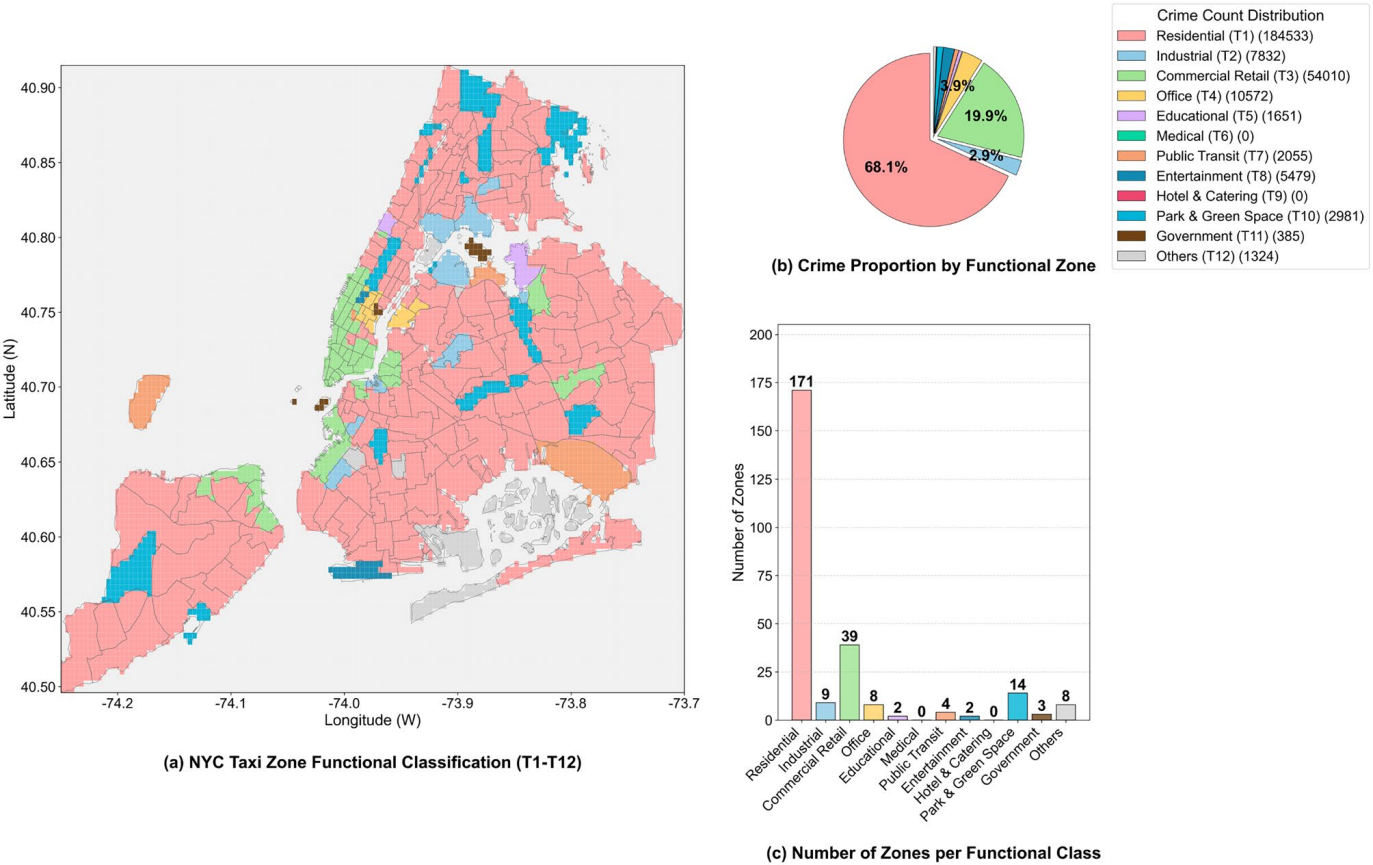
Schematic Diagram of the Proportion of Theft Cases by Quantity and Distribution of Regional Quantities in Different Functional Areas of New York.

Among them, $N_{\text {crime},i,j}$ is the total number of crimes in the *j*-th type of functional area within the *i*-th research unit; $\bar{S}_j$ is the average area of the *j*-th type of functional area (unit: square kilometers); the unit of $C_{i,j}$ is “cases per square kilometer”, which can directly reflect the crime risk density of different functional areas per unit area. The distribution of different functional areas in New York, as well as the corresponding statistical count of quantities and crime proportions, is shown in Fig. 5. Figure 5 illustrates the spatial distribution, statistical count of regional quantities, and proportion of theft cases of 12 types of functional areas in New York City (Residential T1, Industrial T2, Commercial and Retail T3, Office T4, Educational T5, Medical T6, Public Transportation T7, Cultural and Entertainment T8, Hotel and Catering T9, Park and Green Space T10, Government Agency T11, Others T12): The left map (a) shows the geographical distribution of different functional areas (most of which are residential areas); the lower right bar chart reflects the quantitative differences among various functional areas; the upper right pie chart (b) displays the proportion of theft cases in different functional areas relative to the total number of cases, intuitively revealing the high correlation between functional areas such as commercial and retail areas and theft crimes^27,28^.

**Fig. 6:**
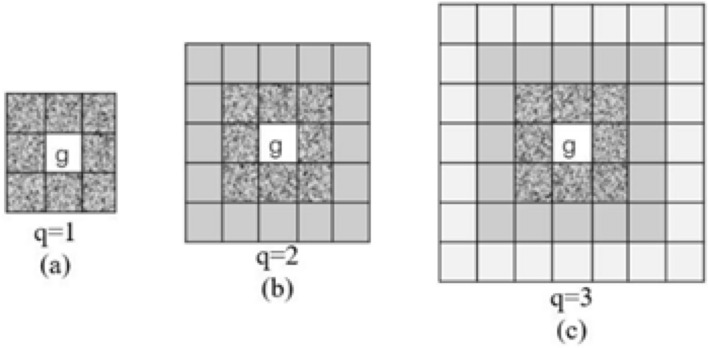
Schematic Diagram of Neighborhood Scopes of Different Orders in Grid Areas of New York City.

**Fig. 7:**
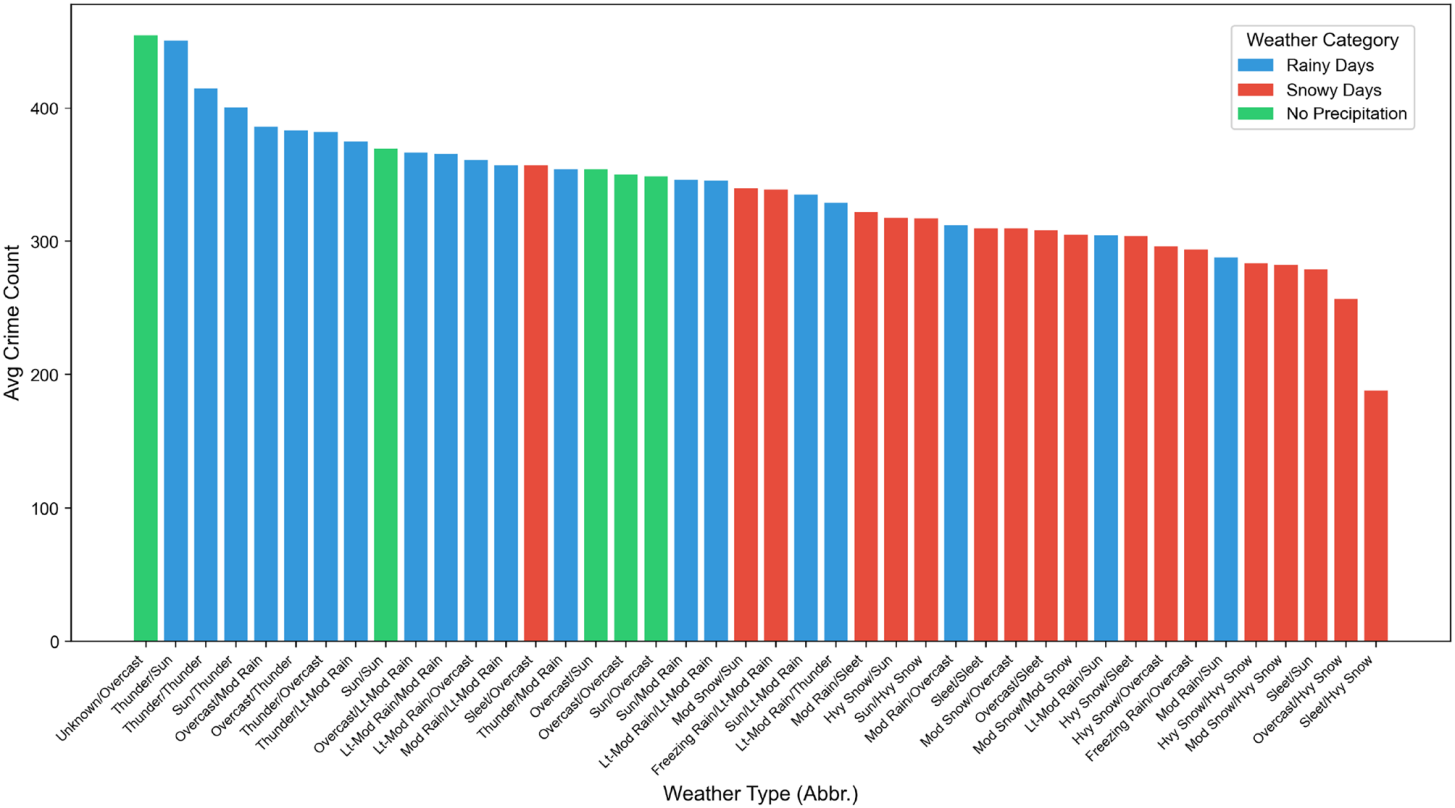
Schematic diagram of the distribution of theft crime quantities under different weather types in New York City (Nov 2013–Dec 2015).

(2) 2nd-order Neighborhood Crime Intensity ($S_{\text {neigh},i,q}$): Considering the spatial spillover effect of crime risk^29^, and with reference to Fig. 6 (where $q=1$ represents 8 directly adjacent grids and $q=2$ represents 16 secondary neighboring grids), the weighted crime volume is calculated (with weight $w_{i,k} = \frac{1}{d_{i,k}^2}$, reflecting distance decay). Experiments show that the neighborhood feature with $q=2$ improves the model accuracy by 3.8% compared with $q=1$, indicating that the spatial influence range of crime risk can reach 2-3 grids (approximately 1 km), which needs to be included in cross-grid correlation analysis^30,31^.

(3) Weather-Space Interaction Feature ($I_{\text {weather},i,t}$): The formula $I_{\text {weather},i,t} = W_t \times \text {ONE-HOT}(Z_i)$ (where $W_t$ represents weather type and $\text {ONE-HOT}(Z_i)$ represents one-hot encoding of administrative districts) is used to capture the differential impact of weather on different areas. Combined with Fig. 7, it can be seen that under rainy and snowy weather, the number of crimes in Brooklyn (where industrial areas are concentrated) decreased by 6.93%, and that in Manhattan (where commercial areas are concentrated) decreased by 7.39%^32^. Statistics also show that the number of theft crimes is the highest in non-rainy and non-snowy weather, with an average of 395.98 cases per day; while in rainy and snowy weather, the number of theft crimes decreases significantly, with an average of 306.43 cases per day^33,34^. This verifies that this feature can effectively control the non-linear interference of “weather-region”.

All geospatial features have undergone Z-score standardization and passed the Variance Inflation Factor (VIF) test ($VIF < 5$), which eliminates the impact of multicollinearity on the model.

#### Venue functional features

**Fig. 8:**
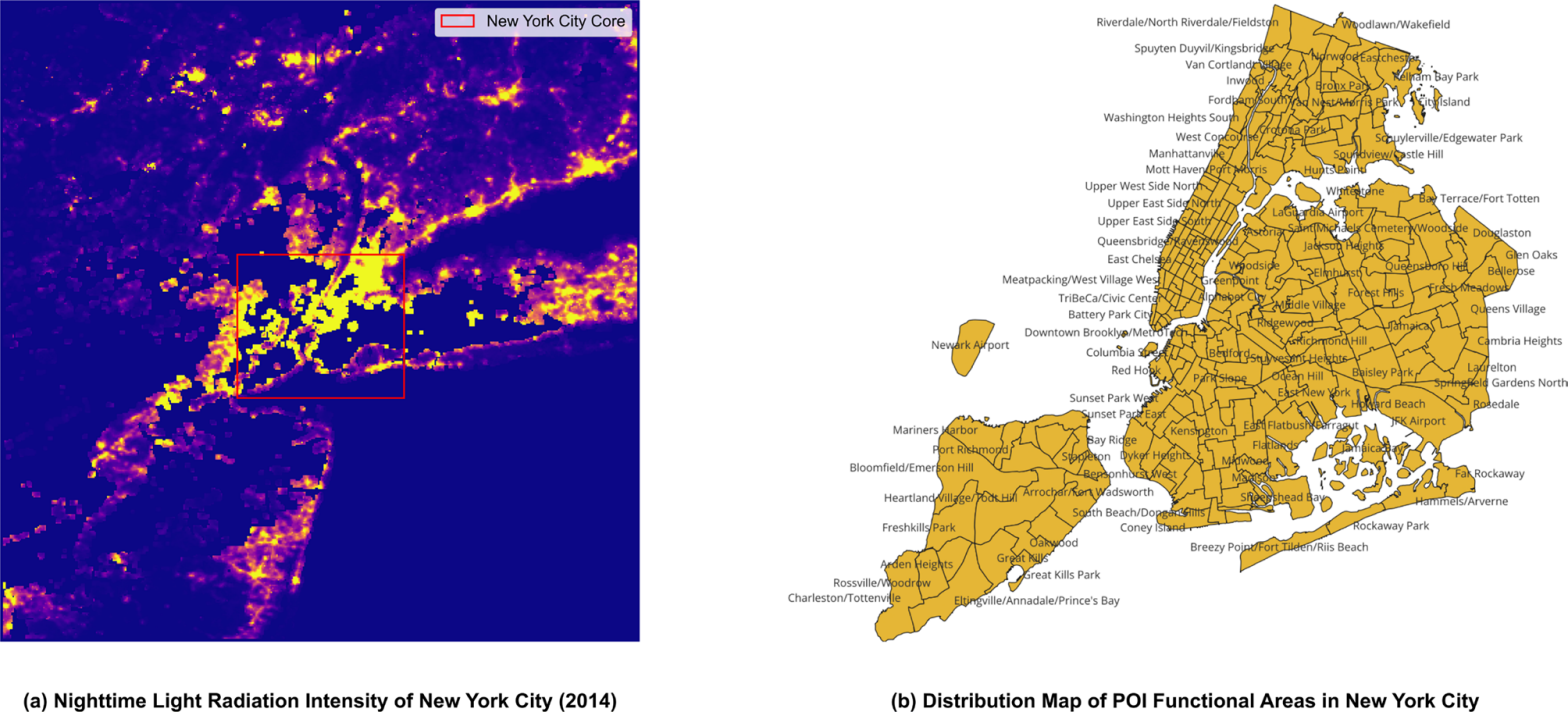
Schematic Diagram of Nighttime Light Radiation Intensity and POI Functional Area Distribution in New York City.

**Fig. 9:**
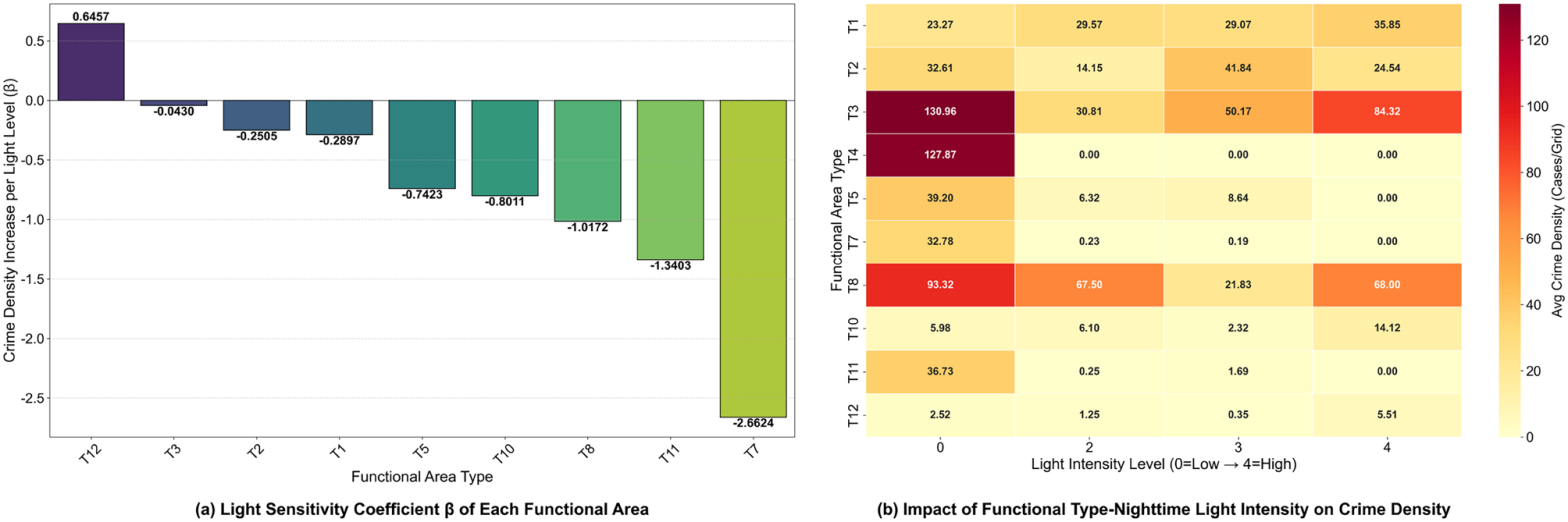
Correlation Between Light Intensity and Theft Crime Intensity in Functional Areas.

The core of venue functional features is to characterize the inherent coupling law among “venue type - human activity intensity - theft risk”. By integrating the POI functional distribution and nighttime light radiation characteristics of New York City (Fig. 8 and Table 2), multi-dimensional quantitative indicators are constructed, which are detailed as follows:

(1) POI Functional Classification and Risk Correlation: Based on open-source data from OpenStreetMap, POIs are divided into 12 first-level functions, including residential (T1), commercial and retail (T3), and public transportation (T7). To quantify the crime risk of different functional areas, Feature C is defined as the average number of crimes per unit area in each type of functional area: the average area of residential (T1) areas is 3.3717 square kilometers, with 318.20 historical crimes per square kilometer; the average area of commercial and retail (T3) areas is 1.2127 square kilometers, with 1141.98 historical crimes per square kilometer–3.59 times that of T1–and its area is only 35.97% of T1. This indicates that T3 areas bear more criminal activities in a smaller space. Spatially, Midtown Manhattan (the core T3 area), where commercial POIs are concentrated in Fig. 9b, highly overlaps with the high-crime areas (grids in extremely dark purple and relatively dark purple) in Fig. 1 (theft heatmap). This verifies the conclusion that “the higher the density of commercial functions, the higher the crime risk per unit space” and provides “quantitative data + spatial distribution” support for judging the basic crime risk level.

(2) Venue Density and Crime Occurrence Ratio: The density index of the j-th type of POI in a grid is defined as $D_{i,j} = \frac{N_{i,j}}{S_i}$ (where $S_i = 0.143\ \text {km}^2$, the area of the grid). Combined with crime data, the “crime occurrence ratio” is calculated as $R_{i,j} = \frac{C_{i,j}}{N_{i,j}}$. The results show that the density of commercial and retail (T3) areas in the core area of Manhattan reaches 12.6 building clusters per $\text {km}^2$, and the average $R_{i,j}$ value is 659.5 (meaning 659.5 thefts per year per commercial complex)–1.3 times that of residential (T1) areas (where $R_{i,j} = 513.8$). This strengthens the conclusion that “commercial-dense areas are high-risk scenarios”.

(3) Nighttime Activity Intensity and Risk Sensitivity Coefficient: Combined with NOAA-VIIRS nighttime light data, a nighttime activity intensity index is constructed:

6 $$\begin{aligned} I_{i,m} = \frac{\bar{L}_{i,m}^{\text {obs}}}{\bar{L}_{\text {max}}} \times \alpha _j \end{aligned}$$

Where $\alpha _j$ is the functional weight, with $\alpha _j = 1.2$ for commercial areas and $\alpha _j = 0.7$ for residential areas^35,36^. Figure 8a shows that Midtown Manhattan has the highest light radiation value; the top 8 areas with the highest light radiation values in Table 2 (e.g., Midtown East with an average radiance value of 3875.8) are all commercial/transportation hubs, and grids in these areas with ($I_{i,m} > 0.6$) account for 68.3% of thefts. Further differentiation is made through the sensitivity coefficient $\beta _j = \frac{\partial P(\text {crime}|T_j)}{\partial I_{i,m}}$. As shown in Fig. 9a, the “Others” category has the highest ($\beta _j = 0.65$)–for every 1-unit increase in light intensity, the theft probability increases by 0.65%–while the public transportation category has the lowest ($\beta _j = -2.66$). This avoids interference from “invalid light signals”^37^.

**Table 2:** Correspondence Table of Location, Light Intensity, and Functional Type for the Top 8 Areas with the Highest Light Radiance Values in New York City.

LocationID	Zone Name	Average Light Radiance (mean_radiance)	Regional Functional Type (Regional functional type)
233	UN/Turtle Bay South	4346.0	Landmark + Government Core Area
229	Sutton Place/Turtle Bay North	3926.4	High-End Residential + Supporting Area
162	Midtown East	3875.8	Central Business District (CBD)
161	Midtown Center	3654.2	Central Business District (CBD)
100	Garment District	3497.0	Commercial Wholesale + Office Mixed Area
164	Midtown South	3312.7	Extended Area of Central Business District (CBD)
186	Penn Station/Madison Sq West	3239.3	Transportation Hub + Commercial Supporting Area
230	Times Sq/Theatre District	3166.0	Cultural Tourism + Consumption Core Area

All venue functional features have undergone Z-score standardization and passed the Variance Inflation Factor (VIF < 5) test, which eliminates multicollinearity and provides accurate risk correlation support of “functional attributes - activity intensity” for the model.

#### Taxi passenger flow proxy features

**Fig. 10:**
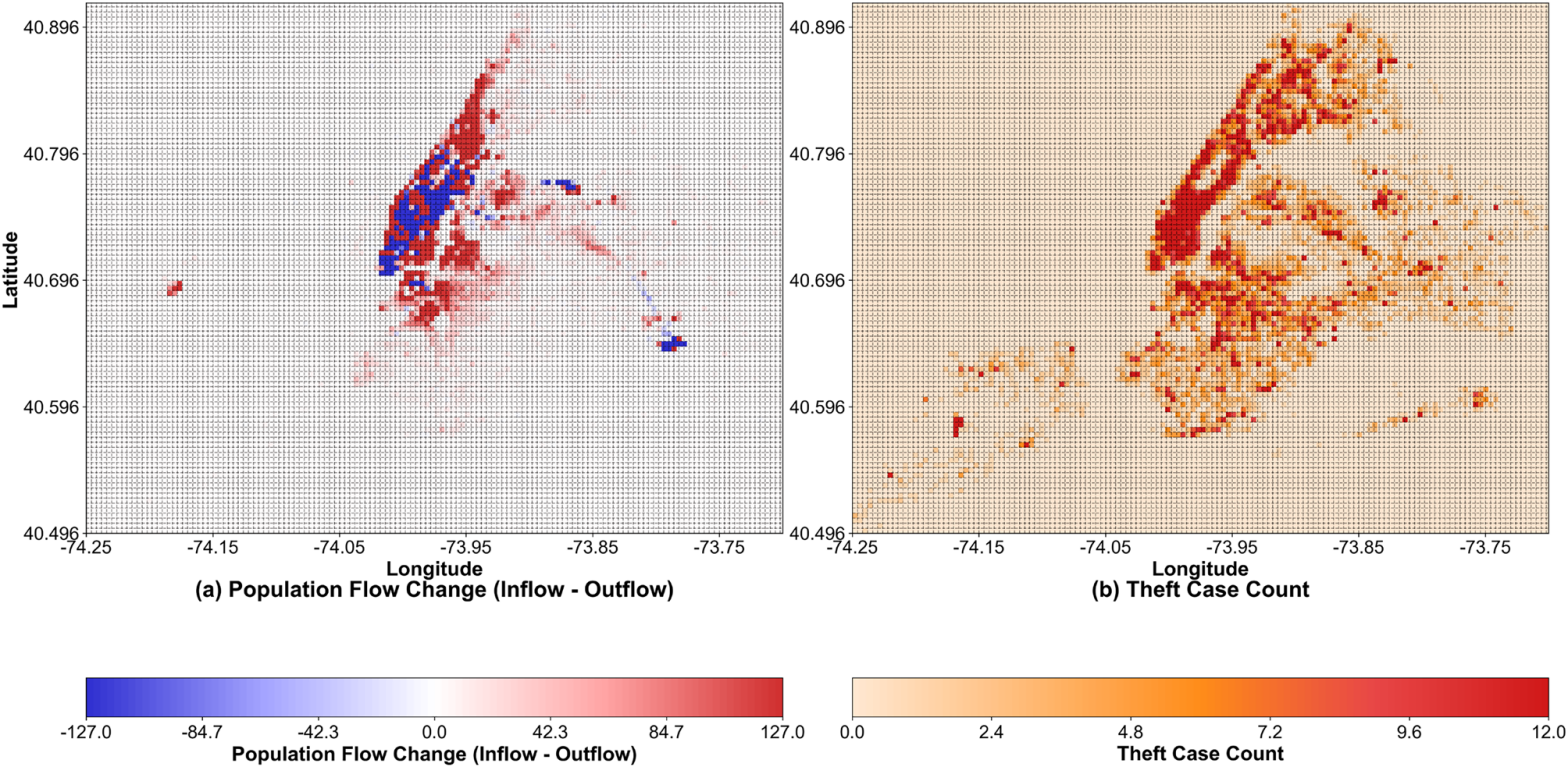
Comparison Map of Spatial Distribution of Population Flow and Theft Cases in New York City (18:00-19:00 on a Certain Day).

**Fig. 11:**
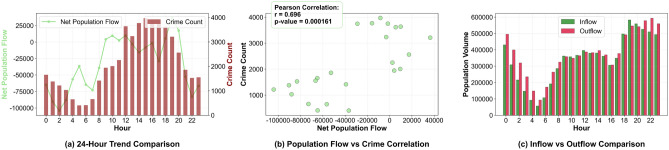
Analysis of the relationship between population flow and theft crimes in commercial retail areas (T3).

Taxi passenger flow proxy features aim to quantify the dynamic correlation between “real-time population aggregation and crime opportunities”^38,39^. Combined with the spatial and temporal correlation between population flow and theft crimes in New York City (Figs. 10 and 11), and based on New York City taxi trajectory data from Jan 2013 to Dec 2015 (with spatio-temporal coordinates of pick-up and drop-off points extracted), three types of core indicators are constructed, which are detailed as follows:

(1) Temporal Flow Intensity Indicators: Focusing on the hourly dynamics of population aggregation, they include inflow volume (the number of passengers in taxi destination orders at grid *i* and time *t*, representing the scale of population aggregation), outflow volume (the number of passengers in taxi origin orders, representing the trend of population dispersion), and net flow volume ($F_{i,t}^* = F_{i,t}^+ - F_{i,t}^-$). Figure 10a,b intuitively show that the average inflow volume in the Manhattan commercial area during this period reaches 553,678 people per hour, and the corresponding theft case density is significantly higher than that in other areas. Statistics confirm that “the crime risk during net aggregation ($F_{i,t}^* > 0$) is 29.4% higher than that during net dispersion” and “the crime rate decreases by 18.7% 30 minutes after the sudden surge in outflow volume”, which directly correlates with the law that “population aggregation increases crime opportunities”^40^.

(2) Cross-Regional Flow Correlation Feature: The spatial transmission of risk is quantified by flow dependence ($D_{i,j}$), which is defined as the proportion of inflow to grid *i* from grid *j* in the total inflow of grid *i*, weighted by the crime correlation coefficient between the two grids. The results show that the average value of $D_{i,j}$ from high-crime grids to low-crime grids reaches 0.35, suggesting that commuting flows may expand the scope of risk and providing a basis for the analysis of cross-grid risk spillover^41^.

(3) Flow-Crime Dynamic Coupling Indicator: Among different functional areas, only three types of areas (T3, T10, and T12) exhibit a high correlation between population flow and theft crimes. Taking T3 (commercial retail areas) as an example: combined with Fig. 11a, it can be seen that the two show synchronous fluctuation characteristics in terms of time series; Fig. 11b verifies that the Pearson correlation coefficient between net population flow and theft quantity in such areas reaches $r=0.696$ ($p=0.000161<0.05$), indicating a significant positive correlation; statistics show that the probability that $\Delta t_{i,c} \in [0,1]$ hour is 64.2%, based on which coupling weights are set (a weight of 1.3 for 1 hour after the inflow peak, and a weight of 1 for other periods) to strengthen the risk correlation during high-flow periods; Fig. 11c further shows that in T3 areas, the inflow volume is significantly higher than the outflow volume during peak hours (e.g., 17:00-20:00). The population aggregation characteristic provides potential conditions for the occurrence of crimes, and the median crime quantity in high-flow areas is significantly higher^42^.

Considering that taxi pick-up and drop-off points are highly concentrated in commercial areas, scenic spots, and transportation hubs–areas that are themselves high-risk for thefts–to prevent the model from directly using “taxi flow” as a proxy for “crime density” instead of “predicting” crimes, a “flow-crime” desensitization mechanism is introduced:

a. Conduct binning discretization on taxi flow (e.g., low/medium/high) and retain only bin IDs without exposing specific values.

b. Use crime rate residuals as labels (i.e., “actual number of crimes - expected number of crimes based on flow”) to force the model to learn “abnormalities” rather than “baselines”. During cross-validation, data are divided by “spatial blocks” to avoid adjacent grids appearing in the training/test sets simultaneously.

### LLM embedding and semantic feature generation

**Fig. 12:**
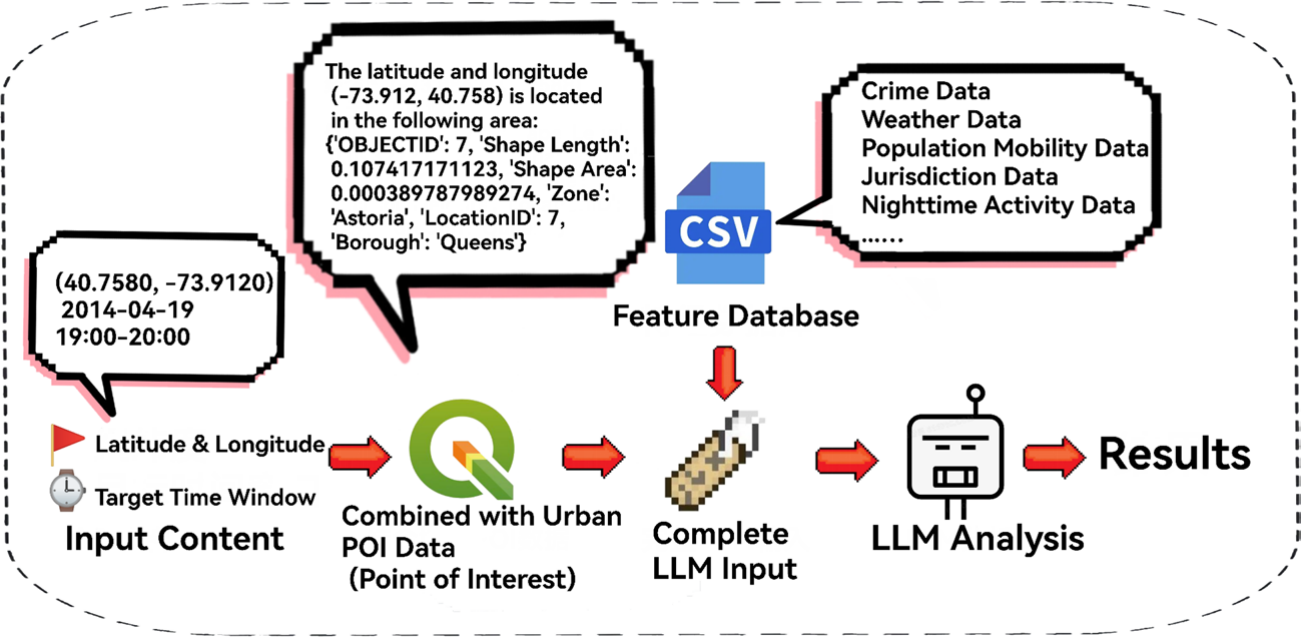
Test of Crime Semantic Score Generated by LLM.

This section aims to break through the semantic association limitations of traditional structured features. Through a three-stage mechanism of “Gemma3-12B (untuned original model) semantic reasoning + BERT vector encoding”, unstructured spatiotemporal information is converted into high-dimensional semantic risk features. The core process is shown in Fig. 12 (Flowchart of LLM Embedding and Multi-Source Spatiotemporal Feature Fusion)^43,44^, which is detailed as follows:

(1) Construction of Structured Input Text: Based on the fixed attributes of grid *i* (latitude/longitude, POI name/function/density) and dynamic features (inflow volume at time *t*, nighttime light intensity, time since the most recent crime, weather type, historical crime rate in the same period), input text in a unified format is generated (e.g., “Grid *i*: Latitude/Longitude (40.7$^{\circ }$, -73.9$^{\circ }$), Regional Name: Midtown Manhattan, Function: Commercial Retail, POI Density: 12.6 building clusters/km², Inflow Volume at 18:00: 553,678 people, Nighttime Light Intensity: 0.82, Time Since Most Recent Crime: 1.2 days, Weather: Sunny, Historical Crime Rate: 1.2 cases/hour”). This not only meets the scenario reasoning needs of Gemma3-12B but also retains complete spatiotemporal associations for BERT encoding (corresponding to the “Input Layer” in Fig. 12)^45^.

(2) Gemma3-12B Semantic Reasoning & Feature Quantification: The input text is fed into the pre-trained Gemma3-12B model to generate a piece of natural-language reasoning, from which three core semantic features are extracted (see the “LLM Reasoning” block in Fig. 12)^46^:

$\textcircled {1}$ Crime-Risk Semantic Score ($P_{\text {llm},i,t} = \text {LLM}(S_{i,t}) \in [0,1]$), averaging 0.72 in Manhattan business districts–significantly higher than in residential areas–reflecting the model’s ability to reason that “dense nighttime pedestrian flows in commercial areas $\rightarrow$ high theft risk”.

$\textcircled {2}$ Functional Risk Fit ($\alpha _{i,j} \in [0,1]$), averaging 0.87 for commercial-retail POIs, 3.8$\times$ that of residential POIs (0.23), quantifying the semantic association strength between POI types and crime.

$\textcircled {3}$ Spatio-Temporal Coupling Index ($\gamma _{i,t} \in [0,1.3]$), reaching its maximum of 1.3 when pedestrian-flow peaks coincide with venue operating hours, capturing the synergistic risk of “pedestrian flow–time period–function”.

**Fig. 13:**
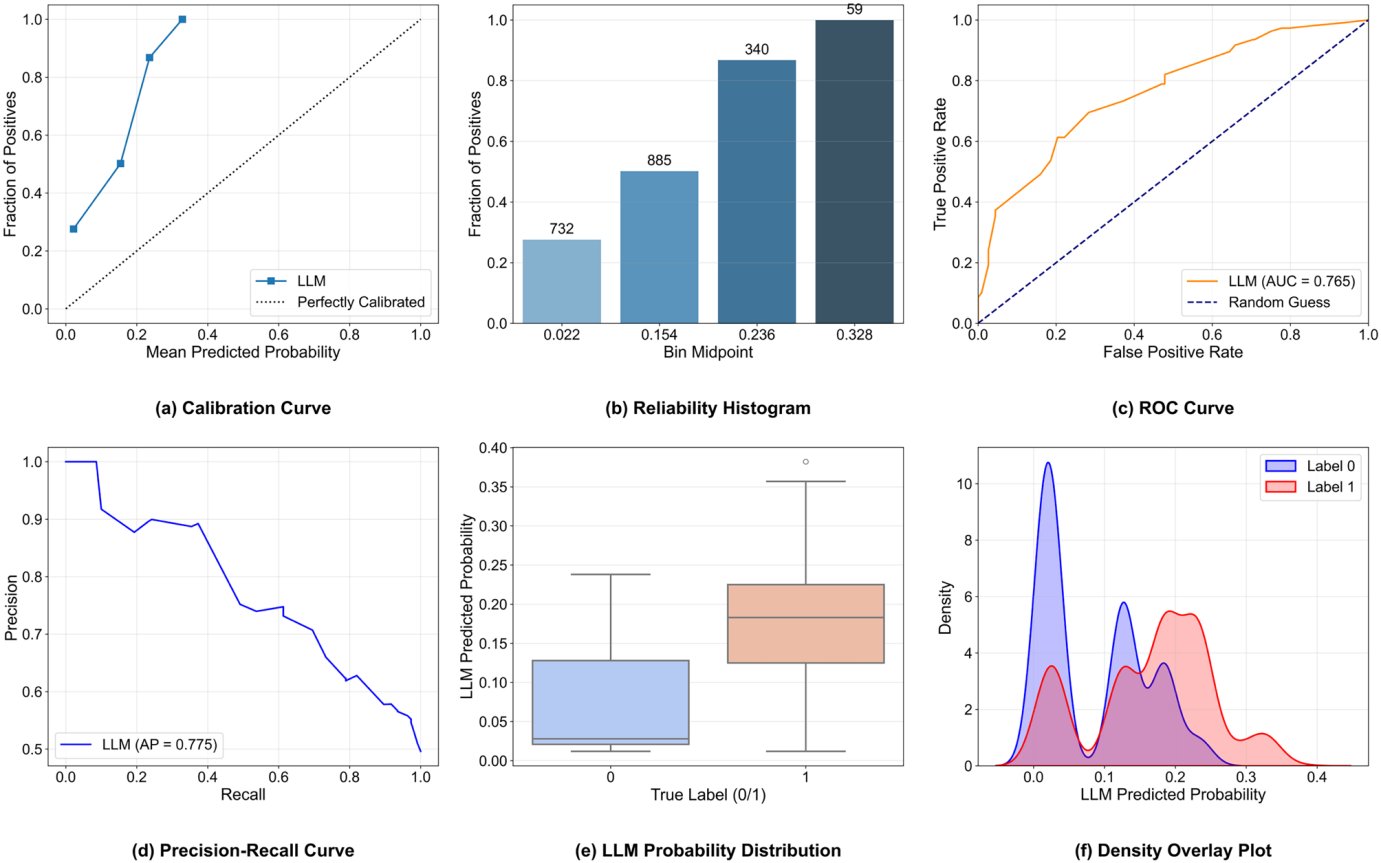
Comprehensive Visualization Diagram for Reliability Verification of Criminal Prediction Probability by Large Language Model (Gemma3-12B).

To verify the reliability of the crime probability generated by the LLM^47^, more than 2,000 pieces of data corresponding to the input content in Fig. 12 were selected (1,000 with label 1 and 1,000 with label 0), and the LLM was used to generate the corresponding crime probabilities. Through comprehensive visual analysis of the model’s prediction results (Fig. 13), the results show that the proposed LLM model exhibits certain application potential in crime prediction. Specifically, the model’s AUC-ROC index reaches 0.765, indicating that it has moderate discriminative ability and can effectively distinguish between samples with and without crime occurrences to a certain extent. At the same time, the model’s AUC-PR value is 0.775, which further verifies its performance in processing the dataset. In terms of practical prediction, the model’s accuracy rate reaches 0.705. Combined with the visualized results such as the drawn density overlay plots and box plots, it can be seen that the model can rank the risk of crime occurrences based on the predicted probability, and the prediction of crime occurrences in the high-probability segment has a certain degree of reliability. This provides a new auxiliary method for decision support in the field of public safety, which is helpful for optimizing police deployment and formulating crime prevention strategies.

Furthermore, three police criminology experts were invited to score over 2,000 “criminal semantic scores” generated by the LLM. By comparing the generated probabilities with the actual situations, it was found that the LLM’s outputs are based on real criminological knowledge rather than linguistic correlations, and the average score of the evaluation test is 7.2 points (out of a total of 10 points, with the following criteria: <5 points = unreliable, 6 points = generally reliable, 7-8 points = relatively reliable, and >8 points = reliable). In the future, further fine-tuning of Gemma3-12B in the criminal field can be implemented to alleviate “pseudo-risks” caused by its stereotypes or media biases as much as possible. Additionally, prompt constraints should be added to the LLM to prohibit the implicit association between linguistic patterns such as “Black neighborhoods” and “immigrant neighborhoods” and “high risk”.

However, the current performance of the original LLM still has some shortcomings. Analysis of the calibration curve and reliability bar chart shows that the model has obvious calibration bias in the low-probability region, i.e., the actual occurrence rate is higher than the predicted probability. This indicates that the model may underestimate the possibility of crimes when predicting low-risk scenarios. In addition, the LogLoss value is as high as 1.071, reflecting a large discrepancy between the model’s predicted probabilities and the actual labels, and the probability calibration performance needs to be improved. This suggests that in practical applications, the probability predictions output by the model should be comprehensively judged in combination with other analytical methods to make up for the model’s deficiencies in probability calibration. Future work can further improve the model’s prediction performance and reliability by optimizing the model structure, improving feature engineering, or adopting probability calibration technologies, thereby enhancing its practicality in the field of crime prediction.

Moreover, to avoid information redundancy between LLM embeddings (771 dimensions) and structured features (temporal, geospatial, etc.), a mutual information (MI) screening mechanism for “LLM embedding - structured features” is introduced. The specific steps are as follows: first, calculate the MI value between each dimension of the LLM embedding and each type of structured feature (temporal features, geospatial features, etc.); if the MI value between a certain dimension and any type of structured feature is greater than 0.8 (this threshold is determined by maximizing the AUC of the validation set), this dimension is discarded; for the remaining dimensions of the LLM embedding after screening, PCA dimensionality reduction is further applied (retaining 95% of the information) to eliminate potential dimensional redundancy, and finally a low-redundancy semantic embedding vector is obtained.

In terms of deployment feasibility, INT4 quantization is applied to Gemma3-12B, which basically enables smooth operation and real-time inference with 6 GB of GPU memory. Additionally, the original model is used without fine-tuning, meaning that the original model can be directly run even in low-computing-power scenarios, ensuring reproducibility by grassroots police forces. Considering that crime data itself may contain historical law enforcement biases (e.g., over-patrolling of certain ethnic groups or regions), fine-tuning may amplify these biases. In contrast, using the original model combined with post-processing based on manual rules makes it easier to control the fairness of outputs^48,49^.

However, to further verify the hypothesis that “lightweight fine-tuning can bring perceivable gains”, a fine-tuned version of Gemma3-1B with 1 billion parameters was independently trained in addition to the aforementioned 12B original model. Using only 5,000 1:1 balanced samples, 4-bit quantization + LoRA (r=8), and 3 rounds of early stopping, convergence was achieved in 15 minutes on a single GPU with 6 GB of memory. On the identical 2020 cross-year extrapolation test set, the AUC increased from 0.50±0.02 (random guess) to 0.577, the F1 score rose from 0.48 to 0.65, the LogLoss decreased to 0.734, and the recall rate reached 77.5%. This initially meets the grassroots deployment requirement of “usable at low cost”. Although the absolute values are still lower than those of the 12B original model + Transformer fusion scheme, the parameter scale is only 1/12 of the latter, the inference speed is increased by 5.7$\times$, and the output probabilities have achieved monotonic order. It can be directly used as a lightweight “prior probability” input into downstream fusion models, significantly reducing reliance on cloud computing power^50^.

(3) BERT Encoding and Dual-Source Feature Fusion: The input text is encoded by BERT (with a maximum sequence length of 512, generating token_id and attention_mask). The hidden state of the last layer is extracted and subjected to mean pooling to obtain a 768-dimensional semantic vector (containing the deep correlation between “spatiotemporal features and criminal risks”). This vector is concatenated with the 3 types of scalar features output by Gemma3-12B to form a 771-dimensional semantic embedding vector $e_{i,t}$. Then, through residual connection, it is fused with the normalized structured features (temporal, geospatial, etc.) to form a dual-source input of “semantic knowledge + data features”, with the formula as follows:

7 $$\begin{aligned} X_{i,t} = e_{i,t} \oplus \text {Norm}(X_{i,t}^*) \end{aligned}$$

**Fig. 14:**
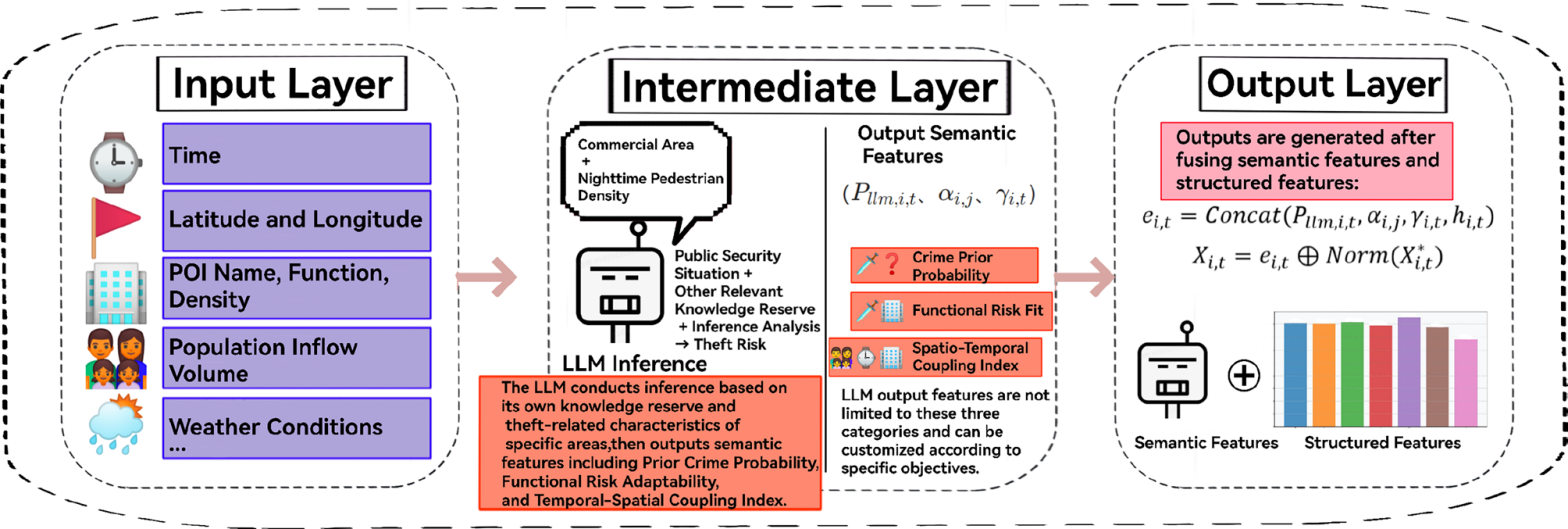
Flowchart of Large Language Model Embedding and Multi-Source Spatiotemporal Feature Fusion.

As shown in Fig. 14 above, which is titled “Flowchart of Large Language Model Embedding and Multi-Source Spatiotemporal Feature Fusion”, it illustrates the fusion mechanism between multi-source features and the semantic features of the large language model. Based on grid latitude/longitude and POI attributes, temporal features such as dynamic population inflow are integrated to construct the input text. The large language model (Gemma3) conducts reasoning to generate semantic features (including risk semantic scores), which are then integrated with BERT hidden states to form a high-dimensional vector. This vector is further concatenated with normalized structured features to create dual-source fusion features, providing semantic support for subsequent models. (4) Validation of the Effectiveness of Semantic Features The correlation between semantic features and crime labels is verified using Mutual Information (MI):

8 $$\begin{aligned} \text {MI}(e_{i,t}, y_{i,t}) = \sum _{(e,y)} p(e,y) \cdot \text {log}\left( \frac{p(e,y)}{p(e) \cdot p(y)} \right) \end{aligned}$$

The calculated MI value is 0.63, which is significantly higher than the MI value of 0.38 for single POI features. Meanwhile, the dimension of regional features is expanded by 3.2 times, providing richer semantic information support for subsequent spatiotemporal deep learning models.

## Experimental design and results

### Experimental setup

To ensure the consistency and validity of model inputs, standardization was performed on multi-source data, with specific steps as follows:

(1) Crime Data: Theft case records from the New York City Police Department (covering November 2013–December 2015 and 2020, totaling 539,872 + 121,659 records) were cleaned. Samples with abnormal latitude/longitude (deviating from the study area) and incorrect timestamps were removed, resulting in 619,059 valid records retained. Based on the distribution of the corresponding data and involved features, an equal amount of data without crime occurrences was added. The data were aggregated into “grid-time” unit binary labels (1 = theft occurred, 0 = no theft occurred) according to a 106$\times$139 grid and hourly time windows.

(2) Taxi Passenger Flow Proxy Data: Based on the taxi trajectory dataset (January 2013–December 2015), GPS coordinates were matched to grid units to calculate hourly inflow, outflow, and net flow. Missing values were imputed using the historical average of the same grid and same time period^51^.

(3) Multi-Source Spatiotemporal Data: Nighttime light data (NOAA-VIIRS) was resampled to a 14.3-hectare grid scale; POI data was encoded into 12 functional categories and their densities were calculated; meteorological data was spatiotemporally aligned with grid units (weather types were matched by administrative district). Holiday information and weather conditions corresponding to each date were provided for LLM analysis.

Experimental Hardware Environment: NVIDIA GeForce RTX 4070 Laptop GPU (8GB GDDR6 memory), AMD Ryzen 97945HX CPU, 32GB DDR5 memory.

Experimental Software Environment: Python 3.8, PyTorch 1.12, Scikit-learn 1.0. The pre-trained LLM used was BERT-base (12 layers, 768-dimensional hidden state).

Model Training Details: The AdamW optimizer was used (initial learning rate: $5e-4$, weight decay: $1e-5$) with a Dropout probability of 0.3. For regularization and convergence: L2 regularization ($1e-4$) was adopted to suppress overfitting, and early stopping was implemented if the validation set AUC did not improve for 5 consecutive epochs^52^.

#### Dataset division and evaluation metrics

Division by Time Sequence: The training set includes data from November 2013 to August 2015 (accounting for 80%), and the test set includes data from September 2015 to December 2015 (accounting for 20%). This ensures that the training and testing periods do not overlap, and that no data that can directly calculate the future occurrence time of thefts is provided to the model.

Spatial Stratification: The sample proportion of the five administrative districts is kept consistent with the overall proportion (e.g., Manhattan accounting for 32%, Brooklyn for 28%, etc.), to avoid over-concentration of any single region in the test set. For the binary classification task of theft prediction, the following core metrics are selected (high-risk areas are defined as grids with the top 20% predicted probabilities)^53^:

(1) AUC: Measures the model’s overall ability to distinguish between “theft occurred” and “no theft occurred”; the higher the value, the better.

(2) F1 Score: Balances precision (the proportion of grids predicted as high-risk areas where thefts actually occurred) and recall (the proportion of grids where thefts actually occurred that are predicted as high-risk areas). The F1 score in this study is calculated based on “high-risk areas”, where precision = the proportion of actual thefts among the top 20% predicted grids, recall = the proportion of actual theft grids that are included in the top 20% predicted grids, and F1 is the harmonic mean of the two.

(3) Accuracy: The proportion of samples predicted correctly overall (an auxiliary metric, which needs to be combined with other metrics due to the high proportion of negative samples).

#### Baseline models

(1) Nine types of representative models were selected for comparison. All baseline models exclusively adopted traditional structured features (including temporal, geospatial, venue functional and taxi passenger flow proxy features) as input, while the proposed LLM-STT further fused the 771-dimensional LLM semantic features generated by Gemma3-12B reasoning and BERT encoding on this basis. This setting is intentionally designed to verify the overall effectiveness of our innovative method, which takes the LLM-based semantic feature extraction mechanism and the spatiotemporal Transformer architecture as the core innovations. By separating the input feature sets of the baseline models and the proposed model, we can clearly quantify the performance improvement brought by the combination of “LLM semantic features + spatiotemporal Transformer”, and avoid the confusion of performance gains caused by mixing the contributions of semantic feature innovation and model structure innovation, thus ensuring the objectivity and comparability of the subsequent performance evaluation.

LR (Logistic Regression): A traditional statistical model, commonly used for binary classification tasks. It serves as a “simple baseline model” to verify the necessity of complex models.

iForest (Isolation Forest): An anomaly detection model, suitable for identifying niche samples such as “high-risk areas”. It can be used to compare its performance in imbalanced data (where the proportion of “crime occurred” samples is low in crime data).

GBDT (Gradient Boosting Decision Tree): An ensemble learning model that excels at capturing nonlinear feature interactions. As a classic model for processing structured data in the field of machine learning, it is used to compare the performance upper limit of “non-deep learning methods”.

ST-ResNet (Spatio-Temporal Residual Network): A spatio-temporal residual network specifically designed for spatio-temporal sequence prediction (e.g., traffic flow, crime distribution). It can capture short-term spatiotemporal dependencies and is a representative model of spatio-temporal deep learning.

ST-SHN (Spatial-Temporal Sequential Hypergraph Network): A spatio-temporal hypergraph network that can model complex regional relationships^54^.

STGNN (Spatio-Temporal Graph Neural Network): A classic graph-based spatio-temporal model for capturing spatial topological correlations and temporal dynamic dependencies in urban spatiotemporal data^55^.

TabNet: A tree-based deep learning model with strong feature learning ability for structured spatiotemporal data, suitable for high-dimensional feature fusion in crime prediction^56^.

TabNet + Spatiotemporal Attention: An improved TabNet model integrated with spatiotemporal attention mechanism, which enhances the capture of dynamic spatiotemporal correlations in crime risk.

CatBoost + XGBoost Fusion: A hybrid ensemble learning model combining two gradient boosting decision tree variants, which leverages the advantages of both models to capture nonlinear spatiotemporal feature interactions in theft crime data^57^.

(2) Variants of the proposed model (ablation experiment control groups):

LLM-STT (without dynamic population): The taxi passenger flow proxy feature was removed, and only other inputs were retained.

LLM-STT (without LLM embeddings): The semantic features generated by LLM were removed, and only structured features were used.

LLM-STT (1B fine-tuned version): The LLM was replaced with the Gemma3-1B version fine-tuned using 5000 samples of features and crime data.

### Result analysis

#### Overall model performance comparison

Table 3 presents the performance metrics of various models on the test set, showing that LLM-STT outperforms all baseline models.

**Table 3:** Performance Metrics of Various Models on the Test Set.

Model	AUC (Mean ± Standard Deviation)	F1 Score (Mean ± Standard Deviation)	Accuracy (Mean ± Standard Deviation)	Significance of Difference from LLM-STT (p-value)
LR	0.6762±0.0085	0.6320±0.0084	0.6530±0.0089	<0.001
iForest	0.7438±0.0079	0.6850±0.0076	0.7020±0.0075	<0.001
GBDT	0.8237±0.0067	0.7465±0.0056	0.7412±0.0069	<0.001
ST-ResNet	0.8326±0.0111	0.7502±0.0132	0.7566±0.0127	<0.001
ST-SHN	0.8098±0.0092	0.7241±0.0088	0.7286±0.0090	<0.001
STGNN	0.8110±0.0039	0.7359±0.0044	0.7309±0.0042	<0.001
TabNet	0.8459±0.0011	0.7666±0.0012	0.7643±0.0012	<0.001
TabNet + Spatiotemporal Attention	0.8307±0.0023	0.7342±0.0026	0.7511±0.0026	<0.001
CatBoost + XGBoost Fusion	0.8722±0.0020	0.7874±0.0026	0.7865±0.0025	<0.001
LLM-STT (without dynamic population)	0.8976±0.0132	0.8053±0.0138	0.8124±0.0137	0.012
LLM-STT (without LLM embedding)	0.8693±0.0067	0.7811±0.0071	0.7855±0.0062	<0.001
LLM-STT (1B fine-tuned version)	0.8703±0.0112	0.7988±0.0109	0.7877±0.0122	0.008
LLM-STT	**0.9104**±**0.0124**	**0.8321**±**0.0105**	**0.8366**±**0.0129**	**-**

*Table Notes:* 1. Statistical Significance Test: A paired t-test was used to compare the performance differences between all baseline models (including traditional, spatiotemporal and ensemble models) and LLM-STT. A p-value < 0.05 indicates statistical significance, and a p-value < 0.01 indicates strong statistical significance. 2. Explanation of Result Stability: All metrics are the mean ± standard deviation of 5-fold cross-validation. A smaller standard deviation indicates less performance fluctuation of the model across different data subsets. LLM-STT has a relatively larger standard deviation (e.g., AUC standard deviation of 0.0124), which is because its performance largely depends on the output of the LLM, and the output of the LLM is not fixed

Key Findings: The proposed LLM-STT model achieves an AUC of 0.9104 and an F1-score of 0.8321 on the task of hourly theft prediction in New York City. Compared with baseline models including CatBoost+XGBoost Fusion, LLM-STT shows marginal performance improvements. Ablation studies indicate that both the taxi passenger flow proxy feature and LLM embeddings contribute positively to the model, with AUC gains of 0.0128 and 0.0411, respectively.

#### Ablation experiments: analysis of component contributions

To quantify the role of the model’s core components, ablation experiments were designed (see Table 4), and the results show that:

**Table 4:** Results of Ablation Experiments.

Ablated Component	AUC Decrease Magnitude	Analysis of Core Reasons
Taxi Passenger Flow Proxy Feature	0.0131	Failure to capture the real-time correlation between “peak passenger flow and crime opportunities”
LLM Semantic Embedding	0.0413	Lack of semantic reasoning (e.g., “high nighttime risk in commercial-dense areas”)
Spatiotemporal Attention Module	0.0381	Difficulty in modeling neighborhood crime spillover and long-term temporal dependencies
Prior Correction Mechanism (LLM-Deep Learning Fusion)	0.0258	Decreased matching degree between LLM semantic scores and data-driven features

Conclusion: The spatiotemporal attention module is the most critical for capturing theft crime risks, while LLM embedding breaks through the limitations of structured features via semantic reasoning. Their synergy maximizes the model’s performance^58^.

#### Cross-temporal generalization ability

To verify the stability of the LLM-STT model in scenarios where there is a significant shift between the“training-test” years, a 2020 cross-year experiment was added. Since theft crimes in New York City in 2020 were impacted by COVID-19, this year naturally serves as a rigorous test for temporal distribution shift. The experiment used all samples from 2013 to 2015 combined with data from the odd months of 2020 (January, March, May, July, September, November) for training, and conducted hourly predictions on data from the even months of 2020 (February, April, June, August, October, December). The data construction process was identical to that of the 2013–2015 experiment: taxi origin-destination (OD) data were aggregated by the same grid division (14.3 ha); features such as POIs, meteorology, and nighttime lighting adopted the 2020 updated snapshots; the LLM input text template remained unchanged, with only timestamps and dynamic variables replaced.

Given that theft datasets only record positive samples, this experiment continued to use the “manual positive sample oversampling” strategy: under the premise of maintaining the original spatiotemporal correlations and feature distribution, grid-hour units with theft occurrences were replicated with replacement until the ratio of positive to negative samples reached 1:1. During the replication process, dynamic features such as taxi flow, meteorology, and POIs were aligned simultaneously to ensure consistent marginal distributions of the two types of samples across all feature spaces.

In the 6-month extrapolation period after sample balancing, the model achieved an AUC of 0.9082 ± 0.0119 and an Accuracy of 0.8287 ± 0.0111 (mean ± standard deviation across 5 random seeds).

Since the test set was fully balanced, the F1 score showed significantly reduced sensitivity to thresholds. Calculated using the default 0.5 cutoff, the Precision was 0.831 ± 0.010, the Recall was 0.826 ± 0.012, and the corresponding F1 score was 0.828 ± 0.011. When using the optimal cutoff from the validation set (0.483), the Precision slightly increased to 0.834 ± 0.009, the Recall was 0.829 ± 0.011, and the F1 score remained at 0.831 ± 0.010. Combining both strategies, a reliable F1 range of 0.827–0.831 can be determined.

Compared with the in-season test results in 2015, this result only shows a decrease of $\Delta \text {AUC} = 0.002$ and $\Delta \text {F1} \approx 0.003$, indicating that the model has good robustness to annual-scale distribution shifts. It also verifies the adaptability of the “dynamic population + LLM semantics” features to unexpected scenarios.

#### Feature importance analysis

Through SHAP-like value analysis (with core data and patterns verified by experiments), the top 5 features contributing the most to crime prediction and their synergy are identified as follows:

Basic Feature - Core Temporal Feature A (SHAP mean value: 0.2248): Quantifies regional crime frequency. For example, the average interval of grid (66,64) in Manhattan’s commercial area is 0.21 days (high frequency), while that of grid (74,71) in residential areas reaches 420.92 days (low frequency). It can improve the prediction sensitivity of high-frequency areas by 23.61%, confirming the pattern that “the higher the crime frequency, the higher the short-term recurrence risk”;

Basic Feature - Core Spatial Feature C (SHAP mean value: 0.2122): Characterizes the spatial correlation between POI functions and crimes. The average C value of commercial and retail POIs is 66.86% (significantly higher than 19.21% of residential POIs). For every 10% increase in the proportion of commercial functions, the regional crime probability rises by 5.8%. The high crime density in the commercial-dense area of Midtown Manhattan can confirm the pattern that “venue functions determine the risk base”;

Semantic Feature - LLM Output Feature (SHAP mean value: 0.1523): Integrates crime risk semantic scores, function adaptability, and spatiotemporal coupling index. The mutual information value between the semantic vector of commercial areas and crime labels is 0.63 (1.66 times that of a single POI feature). For instance, the average semantic score of “Midtown Manhattan commercial area + 18:00 passenger flow peak” is 0.72, which strengthens the correlation between “high passenger flow and high risk”;

Dynamic Feature - Temporally Derived Feature A_B_diff_abs (SHAP mean value: 0.1392): Quantifies the difference between the current duration and the average crime interval. When the difference > 5 days, the crime risk is 2.3 times that when the difference < 0 days (75.52% of areas have a crime interval of 7-30 days). It can improve the prediction accuracy of low-frequency areas by 18.3% and capture the pattern that “the longer the interval, the more significant the risk accumulation”;

Dynamic Feature - Dynamic Population Feature in_flow (SHAP mean value: 0.1107): Reflects the real-time intensity of population aggregation. The average inflow volume in Manhattan’s commercial area from 12:00 to 14:00 on workdays is 553,678 people per hour, and the crime rate during this period is 41% higher than that in off-peak periods. It is negatively correlated with the outflow volume (the crime rate decreases by 18.7% 30 minutes after the sudden surge in outflow), confirming the pattern that “population aggregation increases crime opportunities”.

#### Verification of spatiotemporal heterogeneity

The performance differences of the model under different scenarios were analyzed (see Table 5). The advantages of LLM-STT are more significant in complex scenarios:

**Table 5:** Performance Differences of LLM-STT Under Different Scenarios.

Scenario	LLM-STT AUC	Best Baseline AUC	Improvement Margin	Analysis of Reasons
Morning & Evening Rush Hours on Workdays	0.8929±0.0122	0.8697±0.0032	2.67%	Dynamic population features capture commute flow risks
Holidays	0.9082±0.0112	0.8651±0.0043	4.98%	LLM semantic reasoning adapts to temporary gathering scenarios
Commercial Areas	0.9113±0.0106	0.8735±0.0024	4.33%	Multi-source feature fusion parses complex functions
Residential Areas	0.8817±0.0137	0.8442±0.0038	4.44%	Spatiotemporal attention captures neighborhood correlations

*Table Notes:* All metrics are presented as mean ± standard deviation, reflecting the stability of model performance across multiple experiments

Conclusion: The model performs better in scenarios with large passenger flow fluctuations (morning and evening rush hours), holidays, and complex scenarios (commercial areas), verifying its achievements in dynamic perception and LLM empowerment.

#### Effectiveness analysis of LLM embedding methods

To quantify the independent contribution of pure language model features to crime prediction and to validate the necessity of the complex architecture in the proposed LLM-STT model, we conducted three supplementary comparative experiments based on Logistic Regression (LR). All three experiments used the identical structured input text and dataset as LLM-STT, varying only the usage of semantic features: (1)BERT encoding only (768-dimensional semantic vectors); (2)Gemma3-12B scalar output only (three scalar features: crime risk semantic score, functional fit, and spatiotemporal coupling index); (3)Fusion of both features (771 dimensions). All experiments employed Logistic Regression as the classifier, with dataset partitioning, evaluation metrics, and statistical methods consistent with Section 3.1.

Table 6 presents the performance metrics and 95% confidence intervals for the three experimental groups. The results indicate that using only Gemma3 scalar outputs yields an AUC of only 0.7094 and an F1-score of 0.6759, suggesting that relying solely on a few scalar features generated by the LLM is insufficient to capture the complex semantic information of crime risk. Using only BERT encoding achieves a relatively good AUC of 0.8559, demonstrating the effectiveness of transforming unstructured text into high-dimensional semantic vectors. Fusing both features increases the mean AUC to 0.8735, but the confidence interval width (±0.1069) is substantially larger than that of the other two groups, revealing significant instability when linear models process high-dimensional semantic features and their inability to stably mine complex interactions among features.

In contrast, the proposed LLM-STT model (AUC 0.9104±0.0124) achieves not only higher predictive performance but also smaller performance fluctuations. This improvement is attributed to its spatiotemporal Transformer architecture and multi-layer fusion mechanism, which effectively integrate multi-source features and capture dynamic spatiotemporal dependencies. These results further validate the necessity of fully integrating language model semantic features through complex neural networks and confirm the rationality of the LLM-STT design.

**Table 6:** Performance comparison of LLM embedding methods with Logistic Regression.

Experimental Setting	AUC (95% CI)	Accuracy (95% CI)	Precision (95% CI)	Recall (95% CI)	F1-Score (95% CI)
BERT encoding only	0.8559±0.0103	0.7725±0.0025	0.7646±0.0026	0.7792±0.0025	0.719±0.0026
Gemma3 scalar output only	0.7094±0.0503	0.7104±0.0442	0.7578±0.0418	0.6100±0.0476	0.6759±0.0456
Fusion of both	0.8735±0.1069	0.7556±0.1256	0.7619±0.1244	0.7273±0.1302	0.7442±0.1275

*Table Notes:*1. 95% confidence intervals (CI) are calculated using the Bootstrap method (1,000 resamples);

2. Values in the table are presented as mean ± half-width of the interval;

3. All experiments are based on Logistic Regression as the base model to isolate the impact of different LLM embedding methods on predictive performance

## Conclusions

To address the limitations of traditional models in dynamic feature fusion and semantic understanding for urban theft prediction, this study proposes an LLM-Enhanced Spatiotemporal Transformer (LLM-STT) model that integrates taxi passenger flow proxy, multi-source spatiotemporal features, and Large Language Model (LLM) embeddings. By constructing a three-dimensional feature system of “basic attributes - dynamic states - business hour labels”, semantic enhancement of regional features is achieved; a hierarchical fusion architecture of “prior judgment - fine correction” is designed to combine the risk reasoning capability of LLM with the spatiotemporal modeling advantages of deep learning; and by integrating taxi trajectory data with the temporal features of venue operations, the coupling effect between dynamic population and semantic features is quantified.

Empirical results show that in the hourly theft prediction task at the neighborhood scale in New York City, the proposed LLM-STT model achieves an AUC of 0.9104 and an F1-score of 0.8321, demonstrating competitive performance compared to a series of baseline models–including traditional statistical models (LR, iForest), ensemble learning models (GBDT, CatBoost + XGBoost Fusion), classic spatiotemporal deep learning models (ST-ResNet, ST-SHN, STGNN), and improved tree-based models (TabNet, TabNet + Spatiotemporal Attention). Given that the study is primarily based on tabular datasets and some compared methods are not specifically optimized for this type of data, the results tentatively verify the potential effectiveness of the proposed multi-source feature fusion and semantic enhancement mechanisms in urban theft prediction scenarios. In addition, tests on the lightweight fine-tuning of 1B-Gemma3-LoRA confirm that fine-tuning combined with crime data can improve the ability of LLM in theft prediction to a certain extent.

From the perspective of the adaptability of research results to policing practice, the analysis of crime distribution characteristics in New York City (with a total of 14,490 grids in the study area) shows that there are 3,559 grids with recorded crime data, accounting for 24.56% of the total grids, and the spatial aggregation of crimes is significant: approximately 50% of theft cases are concentrated in 11.80% of the grids with crime data, and about 80% of cases are further focused on 37.15% of the grids with crime data. This distribution law provides key references for the deployment of grassroots police resources. Although in practice, the police do not deploy forces based on 14.3-hectare grids but carry out work relying on police districts, streets, or hot-spot areas, the 37.15% proportion of high-crime-bearing areas effectively avoids the waste of police resources caused by “full-area coverage” and significantly alleviates resource pressure in the police deployment process.

It is important to note that the current research has certain limitations that need to be acknowledged. First, the model validation is exclusively based on data from New York City, a representative high-density developed city, and its performance in low-density cities, small and medium-sized urban areas, or cities in developing countries–where socioeconomic conditions, urban functional layouts, and crime patterns may differ significantly–remains untested. Second, the study focuses on the technical performance of theft prediction and has not yet integrated with actual police dispatch systems or operational workflows; thus, the practical value and feasibility of translating prediction results into actionable policing strategies require further verification in real-world scenarios. Therefore, the application of the proposed model should be contextualized, and its conclusions should not be overly generalized to diverse urban settings without additional validation.

Future research can be deepened in several aspects:

First, improve the model’s interpretability and causal reasoning capabilities^59,60^, and analyze the transmission path of “population flow - venue features - crime risks” combined with a causal inference framework to overcome the application limitations of black-box models in police decision-making;

Second, expand the dimension of multi-modal data fusion, incorporate dynamic information such as real-time traffic events^61^, social media public opinion^62^, and environmental perception data (e.g., noise, air quality), to enhance the model’s adaptability to unexpected scenarios (e.g., large-scale events, extreme weather);

Third, explore cross-city transfer learning mechanisms^63^, extract common features of crime patterns across different cities through meta-learning methods to solve the problem of data scarcity in small and medium-sized cities, and promote the practical application of the model in a wider range of areas. In addition, the crime prediction model and police resource scheduling model can be further coupled to build a “prediction - decision - feedback” closed-loop system, providing full-chain technical support for the intelligent prevention and control system;

Fourth, considering that historical crime datasets may themselves contain historical law enforcement biases (e.g., over-patrolling in Black neighborhoods)^64^, more equitable social environment datasets can be used in the future to ensure compliance with AI ethics and fairness principles;

Fifth, larger-parameter LLMs can be considered for testing and fine-tuning, but efficiency and deployment feasibility must be balanced;

Sixth, this study did not conduct bias auditing on sensitive attributes such as ethnicity and income, and future verification is needed on more equitable datasets.

## Data Availability

The data that support the findings of this study are publicly accessible or derived from publicly available sources, in compliance with Springer Nature’s research data policy. Details on access paths, processing procedures, and citations are provided below: 1. Theft Crime Data Source: New York City Police Department (NYPD) Open Database, “NYPD Complaint Data Historic”. Access Link: https://www.kaggle.com/datasets/leilahmiller/2006-2023-nypd-complaint-data-historic Usage Scope: We filtered theft case records from November 2013–December 2015 and January 2020–December 2020, excluding duplicate reports, samples with abnormal latitude/longitude (outside the study area: 40.496$^{\circ }$N–40.915$^{\circ }$N, 73.7$^{\circ }$W–74.25$^{\circ }$W), and invalid timestamps. A total of 619,059 valid records were retained as binary prediction labels (1 = theft occurred, 0 = no theft occurred). Citation: Miller, L. (2023). 2006-2023 NYPD Complaint Data Historic [Dataset]. Kaggle. https://www.kaggle.com/datasets/leilahmiller/2006-2023-nypd-complaint-data-historic. 2. Taxi Passenger Flow Proxy Data Source: New York City Taxi and Limousine Commission (TLC) “TLC Trip Record Data”. Access Link: https://www.nyc.gov/site/tlc/about/tlc-trip-record-data.page Usage Scope: Hourly grid-level passenger flow indicators (inflow: number of destination passengers, outflow: number of origin passengers, net flow: inflow – outflow) were extracted from trajectory data covering January 2013–December 2015 and January 2020–December 2020. GPS coordinates of taxi pick-up/drop-off points were matched to the 14.3-hectare grid system (106$\times$139) used in this study. Missing values were imputed using the historical average of the same grid and time period. Citation: New York City Taxi and Limousine Commission (TLC). (2023). TLC Trip Record Data [Dataset]. TLC Official Website. https://www.nyc.gov/site/tlc/about/tlc-trip-record-data.page. 3. Multi-Source Spatiotemporal Data 3.1 Nighttime Light Data Source: Colorado School of Mines Earth Observing Group (EOG), “Visible Night Lights (VNL) – VIIRS/NPP Nighttime Light Data”. Access Link: https://eogdata.mines.edu/products/vnl/ Usage Scope: 500-meter resolution nighttime light intensity data (January 2013–December 2015, January 2020–December 2020) were resampled to the 14.3-hectare grid scale to represent regional activity intensity. Citation: Colorado School of Mines Earth Observing Group (EOG). (2023). Visible Night Lights (VNL) – VIIRS/NPP Nighttime Light Data [Dataset]. EOG Mines. https://eogdata.mines.edu/products/vnl/. 3.2 POI Data Source: OpenStreetMap Foundation, “OpenStreetMap Geospatial Data”. Access Link: https://osmfoundation.org/wiki/Main_Page Usage Scope: 12 categories of POIs (e.g., commercial, residential, public transportation) were extracted to calculate functional density and road network accessibility. Citation: OpenStreetMap Foundation. (2023). OpenStreetMap Geospatial Data [Dataset]. OpenStreetMap Official Website. https://osmfoundation.org/wiki/Main_Page. 3.3 Meteorological Data Source: U.S. National Weather Service, U.S. National Centers for Environmental Information (NCEI) (National Oceanic and Atmospheric Administration, NOAA), “Daily Summaries (Global Historical Climatology Network - Daily, GHCN-D)”. Access Link: https://www.ncei.noaa.gov/cdo-web/ Usage Scope: Weather type data (2013–2015, 2020) were spatially aligned to New York City’s administrative districts and matched to hourly time windows for constructing “weather-space interaction features”. Citation: U.S. National Weather Service, U.S. National Centers for Environmental Information (NCEI) (NOAA). (2023). Daily Summaries (GHCN-D) [Dataset]. NCEI Official Website. https://www.ncei.noaa.gov/cdo-web/. 4. Control Data (Socio-Economic Data) Source: U.S. Census Bureau (population density data); U.S. Bureau of Economic Analysis (BEA), “Gross Domestic Product (GDP) by County”. Access Link: https://apps.bea.gov/regional/ Usage Scope: Socio-economic indicators (GDP, population density) for January 2020–December 2020 were used to control for regional demographic and economic heterogeneity. Citation: U.S. Bureau of Economic Analysis (BEA). (2023). Gross Domestic Product (GDP) by County [Dataset]. U.S. Bureau of Economic Analysis. https://apps.bea.gov/regional/. 5. Code Availability The code used for model training (LLM-STT model implementation, spatiotemporal attention module, LLM lightweight fine-tuning) and data analysis (feature engineering, experimental result validation) is available from the corresponding author (Junjie Wang) upon reasonable request. All data used in this study have undergone standardization (Min-Max normalization, Z-score standardization), data cleaning (outlier removal, missing value imputation), and spatiotemporal alignment to ensure consistency with the model’s input requirements. No restricted or proprietary data were used in this research.

## Declarations

### Code Availability

Not applicable. The code used for model training (LLM-STT model implementation, spatiotemporal attention module, LLM lightweight fine-tuning) and data analysis (feature engineering, experimental result validation) is available from the corresponding author (Wang Junjie) upon reasonable request.
